# HPLC method for quantifying verbascoside in *Stizophyllum perforatum* and assessment of verbascoside acute toxicity and antileishmanial activity

**DOI:** 10.3389/fpls.2023.1324680

**Published:** 2023-12-08

**Authors:** Osvaine Junior Alvarenga Alves, Saulo Duarte Ozelin, Larissa Fernandes Magalhães, Ana Carolina Bolela Bovo Candido, Valéria Maria Melleiro Gimenez, Márcio Luís Andrade e Silva, Wilson Roberto Cunha, Ana Helena Januário, Denise Crispim Tavares, Lizandra Guidi Magalhães, Patricia Mendonça Pauletti

**Affiliations:** ^1^ Center for Research in Exact and Technological Sciences, University of Franca, Franca, São Paulo, Brazil; ^2^ Faculty of Animal Science and Food Engineering, University of São Paulo, Pirassununga, São Paulo, Brazil

**Keywords:** antileishmanial, acute toxicity, *Stizophyllum perforatum*, validation, verbascoside HPLC method, antileishmanial activity

## Abstract

We report the chemical composition of the crude leaf extracts obtained from *Stizophyllum perforatum* (Cham.) Miers (Bignoniaceae), a simple high-performance liquid chromatography–diode array detection (HPLC-DAD) method based on mangiferin as an internal standard to quantify verbascoside, and the verbascoside acute oral toxicity and antileishmanial activity. HPLC–high-resolution mass spectrometry–DAD (HPLC–HRMS–DAD) analyses of the crude ethanol *S. perforatum* leaf extracts (CE-1 and CE-2) revealed that verbascoside was the major constituent in both extracts. CE-1 was purified, and verbascoside and casticin, among other compounds, were isolated. The developed HPLC-DAD method was validated and met the required standards. Investigation of the CE-2 acute toxicity indicated a lethal dose (LD_50_) greater than 2,000 mg/kg of body weight. Both CE-1 and CE-2 exhibited antileishmanial activity. The isolated compounds, verbascoside and casticin, also displayed antileishmanial activity with effective concentrations (IC_50_) of 6.23 and 24.20 µM against promastigote forms and 3.71 and 18.97 µM against amastigote forms of *Leishmania amazonensis*, respectively, but they were not cytotoxic to J774A.1 macrophages. Scanning electron microscopy of the *L. amazonensis* promastigotes showed that the parasites became more rounded and that their plasma membrane was altered in the presence of verbascoside. Additionally, transmission electron microscopy demonstrated that vacuoles emerged, lipids accumulated, kinetoplast size increased, and interstitial extravasation occurred in *L. amazonensis* promastigotes exposed to verbascoside. These findings suggest that *S. perforatum* is a promising candidate for further *in vivo* investigations against *L. amazonensis*.

## 1 Introduction

*Stizophyllum perforatum* (Cham.) Miers (Bignoniaceae), synonyms *Bignonia perforata* Cham. and *Bignonia physaloides* Cham., is a liana that grows in Mexico, Guatemala, Costa Rica, Panama, Guyana, and Brazil. The genus *Stizophyllum* comprises only three species, *S. perforatum*, *Stizophyllum inaequilaterum*, and *Stizophyllum riparium*, which are morphologically similar and occur in wet to dry forests and disturbed vegetation from Mexico to southern Brazil. Interestingly, *Stizophyllum* species are pioneers and are typically found in disturbed areas, forest margins, and secondary vegetation (Lohmann and Taylor, 2014; Beyer et al., 2017). Additionally, the crude leaf extract obtained from *S. perforatum* has been shown to exhibit trypanocidal activity (Silva et al., 2014).

Triterpenes and pregnane derivatives with cytotoxic action against P-388 lymphocytic leukemia have been isolated from *S. riparium* whole plant extract (Duh et al., 1987; Duh et al., 1991). In Peru and Panama, *S. riparium* is used as folk medicine to treat snakebites (Valadeau et al., 2010) and to dilate atrophied urethras in cases of anuria (Gupta et al., 1993). However, no information regarding the isolation of compounds from *S. perforatum* or its antileishmanial activity has been published.

The occurrence of phenylethanoid glycosides in Bignoniaceae species is well documented (Alipieva et al., 2014; Alvarenga et al., 2015; Bertanha et al., 2020). In this study, we isolated verbascoside, a phenylethanoid glycoside known for its antioxidant, anti-inflammatory, antineoplastic, wound-healing, neuroprotective, and antileishmanial properties, from *S. perforatum* (Alipieva et al., 2014; Maquiaveli et al., 2016a; Maquiaveli et al., 2017).

To the best of our knowledge, no internal standard-based high-performance liquid chromatography–diode array detection (HPLC-DAD) method is available for quantitatively determining verbascoside in *S. perforatum*. Here, we validated such a method by following the Brazilian Health Surveillance Agency regulations. We also investigated the antileishmanial activity of verbascoside against *Leishmania amazonensis*, as well as the changes that verbascoside elicits in *L. amazonensis* promastigotes. Finally, we assessed toxicological aspects by evaluating acute oral toxicity.

## 2 Materials and methods

### 2.1 Instrumentation, materials, reagents, and standards

The NMR data were acquired using a Bruker Avance DRX-400 or Bruker Avance DRX-500 spectrometer with deuterated methanol or deuterated chloroform (CD_3_OD and CDCl_3_, respectively; Sigma-Aldrich, St. Louis, MO, USA) as solvent. The compounds were purified through preparative HPLC on a Shimadzu LC-6AD chromatograph equipped with either an SPD-M20A DAD or an SPD-20A UV-Vis detector; ODS Luna Phenomenex or Shim-pack columns (250 × 10 mm and 250 × 20 mm, respectively) were used. Verbascoside (**1**) and mangiferin (**IS**) were quantified on a Shimadzu LC-20AD chromatograph connected to an SPD-M20A DAD detector, a CTO-20A column oven, and an SIL-20A-HT autosampler. The HPLC method was developed using an ODS Luna Phenomenex column (5 μm, 250 × 4.60 mm). Ultrapure water was obtained using the Millipore^®^ Simplicity system, while methanol, dimethyl sulfoxide, and acetonitrile were HPLC grade. The reagents included water, acetic acid, methanol, ethyl acetate, hexane, and amphotericin B. Mangiferin (100%) had been previously isolated (Pauletti et al., 2003). The chromatographic supports included silica gel (230–400 mesh, Sigma-Aldrich), octadecyl-functionalized silica gel (200–400 mesh, Sigma-Aldrich), and Sephadex LH-20 (GE Healthcare, Chicago, IL, USA). Column chromatography was conducted using a GE Healthcare peristaltic pump model P-1 and a GE Healthcare automatic collector model FRAC-920. The fractions were concentrated on a Büchi rotary evaporator or a Thermo Scientific Savant SPD1010 SpeedVac. HPLC–high-resolution mass spectrometry–DAD (HPLC–HRMS–DAD) chromatograms of the crude *S. perforatum* leaf extracts were acquired on a Bruker Daltonics micrOTOF-QII-ESI-TOF mass spectrometer coupled to a Shimadzu HPLC LC-20AD chromatography; an ODS Luna Phenomenex column was employed.

### 2.2 Plant materials

*S. perforatum* (Cham.) Miers leaves were harvested in Ribeirão Preto and Pirassununga, São Paulo, Brazil (21°7′55.4″S, 47°43′56.4″W and 21°56′15.3″S, 47°28′26.8″W) in January 2014 and February 2018. A voucher specimen (SPFR 16312) was deposited in the Herbarium of the Department of Biology, Laboratory of Plant Systematics, Faculdade de Filosofia Cências e Letras de Ribeirão Preto, University of São Paulo, Brazil (Herbarium SPFR).

### 2.3 Preparation of extracts, stock solution, standard solutions, and samples

The *S. perforatum* leaf materials from the first and second harvests were dried at room temperature and powdered. The powders (110 g and 665 g) were separately macerated with ethanol (400 mL × 3) through three consecutive extractions, at room temperature; each extraction lasted 72 h. The filtrates were concentrated under reduced pressure, which yielded 31 g and 43 g of the crude ethanol *S. perforatum* leaf extracts labeled CE-1 and CE-2, respectively.

Verbascoside (**1**) and mangiferin (**IS**) solutions at 1.0 mg/mL were prepared in methanol and methanol/dimethyl sulfoxide (9:1, v/v), respectively. Subsequently, stock solutions (500 μg/mL) of **1** and **IS** were prepared in acetonitrile. Seven standard solutions of **1** and **IS**, at 0.8, 2, 5, 10, 25, 50, and 60 µg/mL, were prepared in acetonitrile/water (2:98, v/v) from the **1** and **IS** stock solutions. Additionally, the standard solutions of **1** were spiked with **IS** stock solution (500 µg/mL) to achieve an **IS** concentration of 10 µg/mL.

Samples were prepared from CE-1 or CE-2 by weighing 1.5 mg of extract and dissolving it in 1 mL of methanol in an ultrasonic bath for 10 min. Next, the solutions were centrifuged in an Eppendorf centrifuge at 5,000 rpm for 5 min and transferred to vials.

### 2.4 Isolation of compounds

CE-1 (16 g) was dissolved in methanol/water (2:8, v/v) and extracted with *n*-hexane and ethyl acetate. After the solvents were removed, the *n*-hexane (HF; 3.3 g), ethyl acetate (EAF; 4.8 g), and hydromethanol (HMF; 7.2 g) fractions were obtained.

EAF (2.0 g) was purified on a Sephadex LH-20 column using methanol as eluent, which isolated **1** (81 mg) in subfractions 53 and 54. Penduletin (**2**, 2.2 mg) and casticin (**3**, 5.4 mg) were subsequently isolated from subfractions 63 and 64 through a further preparative thin-layer chromatography (TLC) step that employed a chloroform/methanol solvent system (9:1, v/v) as eluent. The same EAF (2.0 g) was also purified by solid-phase extraction (SPE) with octadecyl-functionalized silica gel to yield four subfractions (EAF-1, methanol/water 3:7, v/v; EAF-2, methanol/water 1:1, v/v; EAF-3, methanol; and EAF-4, ethyl acetate). EAF-3 (0.9 g) was submitted to silica column chromatography with a chloroform/methanol gradient as eluent to give 23 subfractions. Subfraction EAF-3.3 (103 mg) was further purified by preparative HPLC with a methanol/water solvent system (90:10, v/v) at a flow rate of 5.0 mL/min. This purification allowed **3** (22 mg), penduletin 4′-methyl ether (**4**, 3 mg), ursolic acid (**5**, 14 mg), and oleanolic acid (**6**, 7 mg) to be isolated.

HF (3.0 g) yielded mixtures of stigmasterol (**7**) and β-sitosterol (**8**) (106 mg), as well as **5** and **6** (133 mg) after silica gel column chromatography with a chloroform/methanol gradient as eluent.

HMF (7.0 g) was subjected to SPE with octadecyl-functionalized silica gel and methanol/water as eluent to give six subfractions. **1** (40 mg) was obtained from fraction 2 (0.6 g, methanol/water 3:7, v/v) through preparative HPLC with acetonitrile/water/acetic acid (23:75:2, v/v/v) at a flow rate of 4.0 mL/min as mobile phase and detection at λ = 330 and 220 nm. The purity of **1** was determined to be 95.0% by HPLC-DAD, and this compound was used to develop and validate the HPLC-DAD method.

Verbascoside (**1**): orange powder (methanol). ^1^H NMR (CD_3_OD, 400 MHz): δ 7.61 (1H, d, *J* = 16.0 Hz, H-7″′), 7.08 (1H, d, *J* = 2.0 Hz, H-2″′), 6.97 (1H, dd, *J* = 2.0 and 8.0 Hz, H-6″′), 6.80 (1H, d, *J* = 8.0 Hz, H-5″′), 6.72 (1H, d, *J* = 2.0 Hz, H-2), 6.69 (1H, d, *J* = 8.0 Hz, H-5), 6.58 (1H, dd, *J* = 8.0 and 2.0 Hz, H-6), 6.30 (1H, d, *J* = 16.0 Hz, H-8″′), 5.20 (1H, d, *J* = 1.2 Hz, H-1″), 4.94 (1H, m, H-4′), 4.39 (1H, d, *J* = 8.0 Hz, H-1′), 4.07 (1H, m, H-8), 3.94 (1H, m H-2″), 3.83 (1H, t, *J* = 9.2 Hz, H-3′), 3.74 (1H, m, H-8), 3.64 (1H, m, H-6′), 3.62 (1H, m, H-3″), 3.55 (3H, m, H-5′, H-6′, and H-5″), 3.41 (1H, t, *J* = 8.0 Hz, H-2′), 3.31 (1H, m, H-4″), 2.81 (2H, m, H-7), 1.11 (3H, d, *J* = 6.4 Hz, H-6″). ^13^C NMR (CD_3_OD, 100 MHz): δ 168.3 (C, C-9″′), 149.8 (C, C-4″′), 148.0 (CH, C-7″′), 146.8 (C, C-3″′), 146.1 (C, C-3), 144.6 (C, C-4), 131.4 (C, C-1), 127.6 (C, C-1″′), 123.2 (CH, C-6″′), 121.2 (CH, C-6), 117.1 (CH, C-2), 116.5 (CH, C-5″′), 116.3 (CH, C-5), 115.2 (CH, C-2″′), 114.6 (CH, C-8″′), 104.2 (CH, C-1′), 103.0 (CH, C-1″), 81.7 (CH, C-3′), 76.2 (CH, C-2′), 76.0 (CH, C-5′), 73.7 (CH, C-4″), 72.3 (CH_2_ and CH, C-8 and C-2″), 72.0 (CH, C-3″), 70.5 (CH, C-4′), 70.4 (CH, C-5″), 62.3 (CH_2_, C-6′), 36.5 (CH_2_, C-7), 18.4 (CH_3_, C-6″).

Penduletin (**2**): yellow powder (methanol). ^1^H NMR (CD_3_OD, 500 MHz): δ 8.02 (2H, d, *J* = 9.0 Hz, H-2′ and H-6′), 6.92 d (2H, d, *J* = 9.0 Hz, H-3′ and H-5′), 6.78 (1H, s, H-8), 3.97 (3H, s, 7-OCH_3_), 3.83 (3H, s, 6-OCH_3_), 3.80 (3H, s, 3-OCH_3_).

Casticin (**3**): yellow powder (methanol). ^1^H NMR (CD_3_OD, 500 MHz): δ 7.67 (1H, brd, *J* = 8.5 Hz, H-6′), 7.64 (1H, brs, H-2′), 7.08 (1H, d, *J* = 8.5 Hz, H-5′), 6.75 (1H, s, H-8), 3.97 (3H, s, 7-OCH_3_), 3.95 (3H, s, 4′-OCH_3_), 3.83 (3H, s, 6-OCH_3_), 3.81(3H, s, 3-OCH_3_). ^13^C NMR (CD_3_OD, obtained from the ^1^H–^13^C heteronuclear single quantum coherence (HSQC) and ^1^H–^13^C heteronuclear multiple bond correlation (HMBC) experiments): 179.0 (C-4), 159.0 (C-7), 156.5 (C-2), 152.0 (C-9), 150.5 (C-4′), 138.0 (C-3), 132.0 (C-6), 122.0 (C-1′), 120.5 (C-6′), 114.5 (C-2′), 110.5 (C-5′), 106.0 (C-10), 90.5 (C-8), 59.5 (6-OCH_3_), 59.0 (3-OCH_3_), 55.5 (7-OCH_3_), 54.5 (4′-OCH_3_).

Penduletin 4′-methyl ether (**4**): yellow powder (methanol). ^1^H NMR (CD_3_OD, 500 MHz): δ 8.12 (2H, d, *J* = 9.0 Hz, H-2′ and H-6′) 7.10 (2H, d, *J* = 9.0 Hz, H-3′ and H-5′), 6.79 (1H, s, H-8), 3.97 (3H, s, 7-OCH_3_), 3.90 (3H, s, 4′-OCH_3_), 3.83 (3H, s, 6-OCH_3_), 3.81 (3H, s, 3-OCH_3_). ^13^C NMR (CD_3_OD, obtained from the HSQC and HMBC experiments): 179.0 (C-4), 162.0 (C-4′), 159.0 (C-7), 156.5 (C-2), 152.0 (C-9), 138.0 (C-3), 132.0 (C-6), 129.8 (C-2′ and C-6′), 122.0 (C-1′), 113.5 (C-3′ and C-5′), 106.0 (C-10), 90.5 (C-8), 59.5 (6-OCH_3_), 59.0 (3-OCH_3_), 55.5 (7-OCH_3_), 54.5 (4′-OCH_3_).

Ursolic acid (**5**): white powder (methanol). ^1^H NMR (CD_3_OD, 500 MHz): δ 5.22 (1H, brs, H-12), 3.16 (1H, dd, *J* = 11.0 and 4.0 Hz, H-3), 2.20 (1H, d, *J* = 11.5 Hz, H-18), 1.11 (3H, s 3H, H-27), 0.98 (6H, s, H-23 and H-26), 0.97 (3H, d, *J* = 6.5 Hz, H-30), 0.88 (3H, d, *J* = 6.5 Hz, H-29), 0.85 (3H, s, H-25), 0.78 (3H, s, H-24). ^13^C NMR (CD_3_OD, obtained from the HSQC and HMBC experiments): δ 180.0 (C, C-28), 138.0 (C, C-13), 125.0 (CH, C-12), 78.0 (CH, C-3), 55.0 (CH, C-5), 53.0 (CH, C-18), 47.0 (C and CH, C-17 and C-9), 41.5 (C, C-14), 39.5 (C, C-8), 39.0 (CH, CH, and CH_2_; C-19, C-20, and C-1), 38.5 (C, C-4), 37.0 (CH_2_ and C, C-22 and C-10), 33.0 (CH_2_, C-7), 30.5 (CH_2_, C-21), 27.5 (CH_2_, C-15), 27.0 (CH_3_, C-23), 26.5 (CH_2_, C-2), 23.5 (CH_2_, C-16), 23.0 (CH_2_ and CH_3_, C-11 and C-27), 20.5 (CH_3_, C-30), 18.0 (CH_2_, C-6), 16.5 (CH_3_, C-25), 16.1 (CH_3_, C-29), 15.0 (CH_3_, C-24), 14.5 (CH_3_, C-26).

Oleanolic acid (**6**): white powder (methanol). ^1^H NMR (CD_3_OD, 500 MHz): δ 5.23 (1H, brs, H-12), 3.15 (1H, dd, *J* = 11.0 and 4.5 Hz, H-3), 2.86 (1H, dd, *J* = 13.5 and 2.5 Hz, H-18), 1.15 (3H, s, H-27), 0.97 (3H, s, H-23), 0.94 (6H, s, H-25 and H-30), 0.90 (3H, s, H-29), 0.82 (3H, s, H-26), 0.77 (3H, s, H-24). ^13^C NMR (CD_3_OD, obtained from the HSQC and HMBC experiments): δ 180.5 (C, C-28), 143.5 (C, C-13), 122.0 (CH, C-12), 78.5 (CH, C-3), 55.2 (CH, C-5), 48.0 (CH, C-9), 46.5 (C, C-17), 45.5 (CH_2_, C-19), 41.5 (C and CH, C-14 and C-18), 39.0 (C, C-8), 38.5 (C, C-4), 38.0 (CH_2_, C-1), 37.0 (C, C-10), 33.5 (CH_2_, C-21), 32.5 (CH_2_, C-7), 32.2 (CH_3_, C-29), 32.0 (CH_2_, C-22), 30.5 (C, C-20), 27.5 (CH_3_, C-23), 27.2 (CH_2_, C-15), 26.5 (CH_2_, C-2), 25.0 (CH_3_, C-27), 23.0 (CH_2_, C-11 and C-16), 22.7 (CH_3_, C-30), 18.2 (CH_2_, C-6), 16.5 (CH_3_, C-26), 14.9 (CH_3_, C-24), 14.5 (CH_3_, C-25).

Sitosterol (**7**): white powder (ethyl acetate). ^1^H NMR (CDCl_3_, 500 MHz): δ 5.35 (brd, *J* = 4.5 Hz, H-6), δ 3.51 (m, H-3), 0.79–2.02 (m). ^13^C NMR (CDCl_3_, 100 MHz): δ 140.7 (C-5), 121.7 (C-6), 71.8 (C-3), 56.8 (C-14), 55.9 (C-17), 50.1 (C-9), 45.8 (C-24), 42.3 (C-13), 42.2 (C-4), 39.6 (C-12 and C-23), 37.2 (C-1), 36.5 (C-10 and C-20), 34.2 (C-22), 31.9 (C-8), 31.8 (C-7), 31.6 (C-2), 28.9 (C-16), 27.9 (C-25), 24.3 (C-15), 22.7 (C-28), 21.0 (C-11), 19.8 (C-27), 19.4 (C-19), 18.9 (C-21 and C-26), 12.2 (C-29), 12.0 (C-18).

Stigmasterol (**8**): white powder (ethyl acetate): ^1^H NMR (CDCl_3_, 500 MHz): δ 5.35 (brd, *J* = 4.5 Hz, H-6), 5.14 (dd, *J* = 8.6 and 15.2, H-22), 5.00 (dd, *J* = 8.6 and 15.2, H-23), δ 3.51 (m, H-3), 0.79–2.02 (m). ^13^C NMR (CDCl_3_, 100 MHz): δ 140.7 (C-5), 138.3 (C-22), 129.2 (C-23), 121.7 (C-6), 71.8 (C-3), 56.8 (C-14), 55.9 (C-17), 51.2 (C-24), 50.1 (C-9), 42.3 (C-13), 42.2 (C-4), 40.5 (C-20), 39.6 (C-12), 37.2 (C-1), 36.5 (C-10), 31.9 (C-8 and C-25), 31.8 (C-7), 31.6 (C-2), 28.9 (C-16), 25.4 (C-28), 24.3 (C-15), 21.2 (C-21), 21.0 (C-11), 19.8 (C-26), 19.4 (C-19), 19.0 (C-27), 12.2 (C-18 and C-29).

### 2.5 HPLC–HRMS analyses

CE-1 and CE-2 at 2 mg/mL were dissolved in methanol and analyzed by HPLC–HRMS. The conditions employed here were the same conditions described by Bertanha et al. in 2020. The HRMS data were obtained using the following conditions: capillary voltage = 3.5 kV, dry temperature = 220°C, nebulizer gas at 60 psi, dry gas at a flow rate of 10 L/min, mass range = 50–1,300 Da, and drying, nebulizing, and collision gas = nitrogen. The HPLC method consisted of a linear gradient of 0.1% acetic acid in water (solvent A) and methanol (solvent B), starting from 95% solvent A and 5% solvent B and going to 100% solvent B over 35 min, followed by 10 min with 100% solvent B; the flow rate was 1.0 mL/min.

### 2.6 Validation

The chromatographic conditions were established through trial and error. Various gradient elution conditions were tested primarily by altering the solvent composition and using CE-2. The optimal conditions were determined using an ODS Luna Phenomenex column (5 μm, 250 × 4.60 mm). Separation was achieved through gradient elution with solvent A (2% aqueous acetic acid) and solvent B (acetonitrile). The following linear gradient was applied: 2%–25% B over 40 min, followed by 25%–100% B in 5 min and 100% B for 5 min. Sample volumes of 20 µL were injected at a flow rate of 1.0 mL/min; the oven temperature was 40°C. The column was equilibrated at 2% B for 15 min. The UV-Vis spectra were recorded from 200 to 600 nm, and the chromatograms were monitored at λ = 330 nm.

The method was validated by following the guidelines of the Brazilian Health Surveillance Agency (ANVISA) and involved assessing several parameters, including selectivity, linearity, limit of detection (LOD), limit of quantification (LOQ), accuracy, and precision (ANVISA, 2017). Selectivity was confirmed using a diode array detector, which allowed the UV-Vis spectra to be obtained and peak purity to be assessed during analyses of the crude ethanol *S. perforatum* leaf extracts. This also helped to verify the identity of the standard by comparing its retention time with the verbascoside standard retention time. To establish linearity, 20 µL of standard solutions of **1** at concentrations ranging from 0.8 to 60 μg/mL and containing 10 µg/mL **IS** was injected into the chromatographic system. A similar procedure was followed for **IS**. Linear regression analysis was applied to the obtained peak areas, and the analyses were performed in triplicate. The linear regression equation of the calibration curve was used to determine LOD and LOQ. Accuracy and precision were assessed using **IS**. CE-2 (1.5 mg in 1 mL of methanol) was spiked with **IS** at 50, 10, or 3 μg/mL, which was followed by sonication. After 10 min, the solution was centrifuged in an Eppendorf centrifuge 5804 at 5,000 rpm for 5 min and transferred to a vial. Accuracy was evaluated by back-calculation and expressed as the percent deviation between the experimental amounts of **IS** and the amounts added at the three examined concentrations. Precision was estimated using relative standard deviation (RSD). Repeatability and intermediate precision tests were conducted on a single day and three non-consecutive days using the samples from the recovery experiment.

### 2.7 Acute toxicity test

Female Wistar Hannover rats (*Rattus norvegicus*) aged between 8 and 12 weeks were obtained from the Animal Facility at the University of São Paulo in Ribeirão Preto. The animals were kept in individual isolators under controlled temperature (23°C ± 2°C) and humidity (50% ± 10%), under a 12-h light–dark cycle, with *ad libitum* access to water and food. The CE-2 acute toxicity and LD_50_ were obtained by following OECD 425 (2022), which classifies compounds in a Global Harmonized System (GHS). CE-2 was diluted in dimethyl sulfoxide and orally administered via gavage; each animal received a single dose of 2,000 mg of CE-2/kg of body weight. Then, the animals were observed for 14 days to monitor possible deaths and behavioral changes. Each animal had its body mass measured each week starting from the oral administration. At the end of the experimental period, the animals were euthanized through intraperitoneal injection of sodium thiopental (Thiopentax 1.0 g; 840 mg/kg of body weight), and a necropsy was conducted to search for macroscopically detectable pathologies. Following macroscopic pathological necropsy, each animal had the liver and kidneys removed for histopathological analysis to evaluate ulceration, necrosis, angiogenesis, and inflammatory infiltrate in the connective tissue, including polymorphonuclear cells like neutrophils, eosinophils, and basophils, as well as mononuclear cells such as monocytes and lymphocytes. The intensity scores 0 (absent), 1 (mild), 2 (moderate), and 3 (intense) were used. Connective tissue fibroplasia was also assessed.

### 2.8 *In vitro* antileishmanial activity

The antileishmanial activity was evaluated against *L. amazonensis* (IFLA/BR/67/PH8) promastigotes following a previously established reference (Alvarenga et al., 2021). For this purpose, *L. amazonensis* promastigotes maintained in RPMI 1640 medium supplemented with 10% fetal bovine serum (FBS) and 1% antibiotic (10,000 IU/mL penicillin and 10,000 mg/mL streptomycin) (Cultilab) were transferred to a 96-well plate (1 × 10^6^). The tested sample (CE-1, CE-2, HF, EAF, HMF, EAF-1, EAF-2, EAF-3, EAF-4, **1**, or **3**) was initially added at 50 µg/mL or 50 µM. Subsequently, promising samples were further evaluated at concentrations ranging from 50 to 3.12 µM or 50 to 3.12 µg/mL. The *L. amazonensis* promastigote cultures were incubated in a biochemical oxygen demand (BOD) incubator at 24°C for 24 h, and the antileishmanial activity was determined by counting the total number of live *L. amazonensis* promastigotes in a Neubauer chamber. The positive and negative controls consisted of amphotericin B (at concentrations ranging from 0.0027 to 1.56 μM) and RPMI 1640 medium containing 0.1% dimethyl sulfoxide, respectively.

The antileishmanial activity was also assessed against *L. amazonensis* amastigotes; the method described by Oliveira et al. (2022) was followed. Briefly, a suspension of 2 × 10^5^ J774.1 macrophages in supplemented RPMI 1640 medium (Gibco^®^) was seeded on glass coverslips in a 24-well microplate and incubated at 37°C under 5% CO_2_ for 2 h. After incubation, the adherent macrophages were infected with *L. amazonensis* promastigotes at a concentration of 1 × 10^6^ cells/well (10:1 ratio) at 37°C for 4 h. Non-internalized *L. amazonensis* promastigotes were washed, and the infected cultures were incubated with one of the tested samples at concentrations ranging from 3.12 to 50 µg/mL or 3.12 to 50 µM for 48 h. Amphotericin B (0.095 to 1.56 μM) and RPMI 1640 medium containing 0.1% dimethyl sulfoxide served as the positive and negative controls, respectively. Next, coverslips were prepared and examined under an optical microscope (Nikon) by counting 200 macrophages to determine the number of *L. amazonensis* amastigotes within each infected cell. In both experiments, the 50% effective concentration (EC_50_) values were determined by non-linear regression analysis. The experiments were performed in triplicate and repeated twice.

Transmission electron microscopy (TEM) and scanning electron microscopy (SEM) analyses were performed by following the protocols of Alvarenga et al. (2016) and Pereira et al. (2015) with some modifications. *L. amazonensis* promastigotes (1 × 10^6^/mL) were incubated with **1** at 8.92 or 89.7 μM (EC_50_ and EC_90_), RPMI 1640 containing 0.1% dimethyl sulfoxide (negative control), or amphotericin B (positive control) at 24°C for 24 h. After incubation, *L. amazonensis* promastigotes were centrifuged and fixed with 3% glutaraldehyde in phosphate buffer (0.1 M, pH 7.2) at 37°C for 1 h and at room temperature for 1 h. The solution was removed, and *L. amazonensis* promastigotes were washed twice with 0.1 M phosphate buffer and processed for TEM or SEM analyses. The samples were analyzed using a JEOL Model JEM-100CXII transmission electron microscope (Tokyo, Japan) or a Joel JSM-5200 scanning electron microscope (Tokyo, Japan) at the Multi-User Microscopy Laboratory of the Ribeirão Preto Medical School.

### 2.9 Cytotoxic activity

The cytotoxic activity of the samples was assessed using the murine macrophage cell line (J774A.1) cultivated in RPMI 1640 medium. The cytotoxicity assay used 3-(4,5-dimethylthiazol-2-yl)-2,5-diphenyl-2H-tetrazolium bromide (MTT; Sigma-Aldrich); the protocol described by Valerino-Díaz et al. (2020) was followed. The macrophages were seeded at 2 × 10^5^ cells/well in 96-well culture plates and incubated at 37°C under 5% CO_2_ for 24 h. Following incubation, the tested sample was added to the plate at final concentrations ranging from 100 to 3.12 µg/mL or µM. The positive control contained 25% dimethyl sulfoxide, while the negative control contained 0.1% dimethyl sulfoxide. The plates were further incubated for 48 h. The concentration that reduced cell viability by 50% (CC_50_) was determined through non-linear regression curve analysis. The experiments were performed in triplicate and repeated twice.

## 3 Results

### 3.1 Isolation and identification of compounds

CE-1 exhibited antileishmanial activity and was initially processed by liquid–liquid partition. At 50 µg/mL, the three resulting fractions showed activity against *L. amazonensis*. However, HF and EAF had stronger activity than HMF, so they were used in further purification steps. EAF was submitted to sequential re-fractionation on a Sephadex LH-20 column to isolate verbascoside (**1**). Subfractions 63 and 64 were then purified by preparative TLC to isolate penduletin (**2**) and casticin (**3**). Another EAF aliquot was processed by SPE to obtain four SPE subfractions (EAF-1, EAF-2, EAF-3, and EAF-4). Bioassays against *L. amazonensis* revealed strong activity for EAF-3, so it was purified by silica gel column chromatography and preparative HPLC to isolate casticin (**3**), penduletin 4′-methyl ether (**4**), ursolic acid (**5**), and oleanolic acid (**6**). HF was purified on a silica gel column to give mixtures of stigmasterol (**7**) and β-sitosterol (**8**), as well as **5** and **6**. HMF also exhibited antileishmanial activity, albeit lower. Nevertheless, it was purified by SPE to isolate **1** in fraction 2 after a preparative HPLC step.

**Figure 1:**
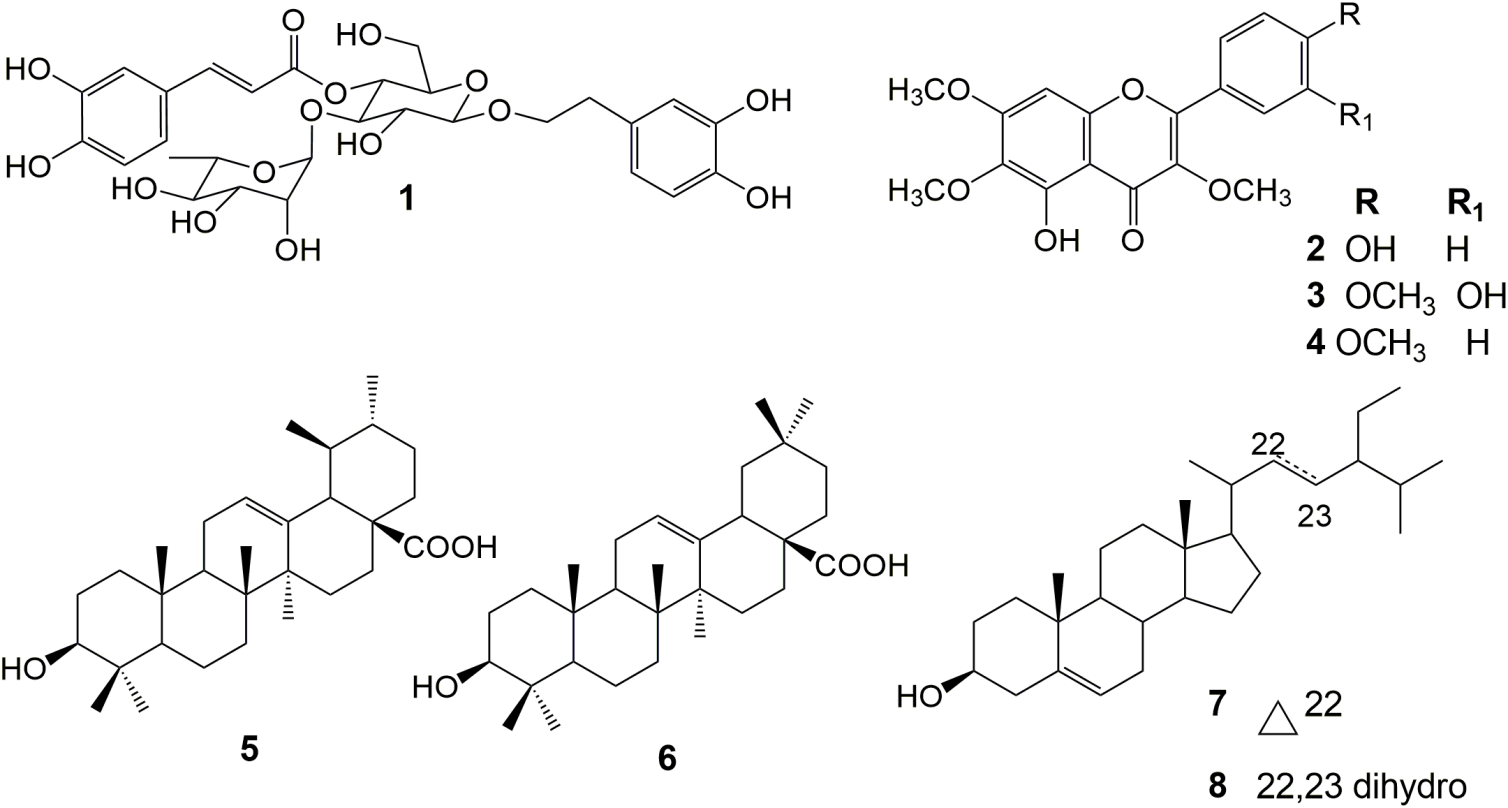
Chemical structure of the isolated compounds.

The spectral data (**Supplementary Material**) of the compounds (**Figure 1**) were consistent with previously published data and allowed us to identify **1**, **2**, **3**, **4**, **5**, and **6** and the mixture of **7** and **8** (Wang et al., 1989; Goulart et al., 1993; Kanchanapoom et al., 2002; Lima et al., 2003; Citoglu et al., 2004; Brandão et al., 2013; Sevindik et al., 2015).

### 3.2 Phytochemical analyses by HPLC–HRMS–DAD

**Figure 2:**
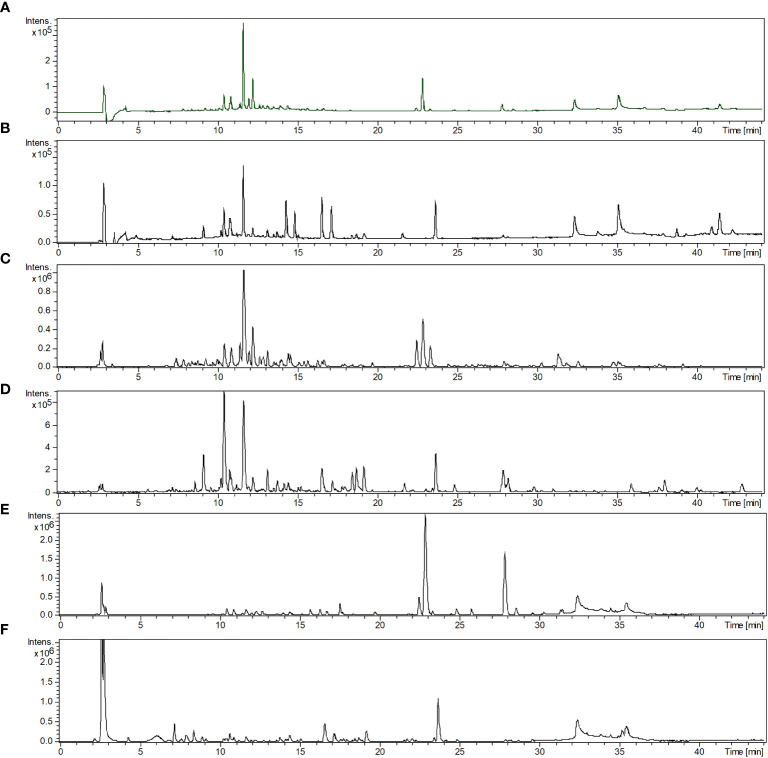
**(A, B)** Chromatograms recorded with UV-Vis detection from 200 to 800 nm. **(C, D)** Base peak chromatogram in the negative mode. **(E, F)** Base peak chromatogram in the positive mode of crude ethanol *Stizophyllum perforatum* leaf extracts CE-1 and CE-2. Analytical conditions: methanol/water (+0.1% acetic acid) linear gradient from 5 to 100% methanol for 35 min and 100% methanol for 10 min. The flow rate was 1.0 mL/min, and the ODS column was Phenomenex Luna.

We conducted phytochemical fingerprinting of CE-1 and CE-2 by HPLC–HRMS–DAD; we employed electrospray ionization in the positive and negative modes, and we set UV-Vis at 200–800 nm. We obtained the chromatograms using a linear gradient of methanol/water (+0.1% acetic acid) from 5% to 100% methanol for 35 min and 100% methanol for 10 min; the flow rate was 1.0 mL/min. The data provided information about the similar chemical profile of *S. perforatum* CE-1 and CE-2 and helped us to select the chemical marker. The DAD detector provided chromatograms (**Figures 2A, B**) for CE-1 and CE-2; **Figures 2C–F** present the total ion chromatogram (TIC) of CE-1 and CE-2 in the negative and positive modes. Combined information from the UV-Vis and mass spectra revealed nine main peaks: at R_t_ of 10.5, 10.9, 11.7, 12.0, 12.3, 22.5, 22.9, 23.4, and 27.9 min for CE-1 and at R_t_ of 9.2, 10.5, 10.8, 11.7, 14.2, 14.9, 16.6, 17.2, and 23.7 for CE-2. **Table 1** describes the possible molecular formulas of the most intense ions with a mass error lower than 5 ppm. Both CE-1 and CE-2 presented peaks at 10.5 and 11.7 min, respectively, which were assigned to C_21_H_20_O_11_ and C_29_H_36_O_15_, respectively. The peak at 11.7 min confirmed that **1** was present in the TIC of CE-1 and CE-2 in the negative and positive modes. Mass spectrometry (**Supplementary Material**) helped to identify the structures of **2** and **3** in CE-1 and CE-2 and flavonoid **4** in CE-1 only.

**Table 1:** Major compounds identified by high-performance liquid chromatography–high-resolution mass spectrometry (HPLC–HRMS) data of crude ethanol *Stizophyllum perforatum* leaf extracts CE-1 and CE-2.

R_t_ (min)	Sample	*m*/*z* [M–H]^−^	*m*/*z* [2M–H]^−^	*m*/*z* [M+H]^+^	*m*/*z* [M+Na]^+^	*m*/*z* [2M+Na]^+^	Suggested formulas	Error (ppm)	Identification
9.2	**CE-2**	325.0918 n. o.	651.1921 n. o.	n. o. 165.0548	349.0897 n. o.	n. o. n. o.	C_15_H_18_O_8_ C_9_H_8_O_3_	1.54, 0.62, 0.57 –2.42	n.i n.i.
10.5	**CE-1**	447.0907	895.1889	449.1068	n.o.	n.o.	C_21_H_20_O_11_	4.47, 4.92, 3.56	n.i.
	**CE-2**	327.1075 447.0914	655.2227 n. o.	n.o. 449.1065	n.o. n. o.	n.o. n. o.	C_15_H_20_O_8_ C_21_H_20_O_11_	1.53, 1.68 -2.91, 4.23	n.i. n.i.
10.8	**CE-2**	325.0913 447.0915 n.o. n.o.	651.1906 895.1914 n.o. n.o.	n.o. n.o. 165.0544 465.1010	n.o. n.o. n.o. n.o.	n.o. n.o. n.o. n.o.	C_15_H_18_O_8_ C_21_H_20_O_11_ C_9_H_8_O_3_ C_21_H_20_O_12_	3.08, 2.92, 2.67, 2.12 4.84 4.94	n.i. n.i. n.i. n.i.
10.9	**CE-1**	447.0913	895.1893	n. o.	n.o.	n.o.	C_21_H_20_O_11_	3.13, 4.47	n.i.
11.7	**CE-1**	623.1951	1,247.3986	625.2115	n.o.	n.o.	C_29_H_36_O_15_	4.01, 3.53, 2.72	Verbascoside*
		n.o. n.o.	n. o. n.o.	433.1123 325.0907	n.o. n.o.	n.o. n.o.	C_21_H_20_O_10_ C_15_H_16_O_8_	2.77 4.92	n.i. n.i.
	**CE-2**	623.1963 n.o. 431.0964	1,247.3995 n.o. n.o.	625.2105 163.0387 433.1120	n.o. n.o. n.o.	n.o. n.o. n.o.	C_29_H_36_O_15_ C_9_H_6_O_3_ C_21_H_20_O_10_	2.08, 2.80, 4.32 4.90 -3.24, 3.46	Verbascoside^a^ n.i. n.i.
12.0	**CE-1**	463.0871 447.0908	n. o. n. o.	n. o. n. o.	n.o. n.o.	n.o. n.o.	C_21_H_20_O_12_ C_21_H_20_O_11_	1.30 4.25	n.i. n.i.
12.3	**CE-1**	623.1951 461.0713	n. o. 923.1501	n. o. n.o.	n.o. n.o.	n.o. n.o.	C_29_H_36_O_15_ C_21_H_18_O_12_	4.01 1.52, 1.84	n.i. n.i.
14.2	**CE-2**	629.2638 n. o. n. o.	n. o. n. o. n. o.	n. o. 197.1173 447.1293	n. o. n. o. n. o.	n. o. n. o. n. o.	C_26_H_46_O_17_ C_11_H_17_O_3_ C_22_H_23_O_10_	3.02 2.53 -0.45	n.i. n.i. n.i.
14.9	**CE-2**	n. o. n. o.	n. o. n. o.	556.3142 679.1628	n. o. n. o.	n. o. n. o.	C_28_H_46_NO_10_ C_41_H_27_O_10_	-3.59 -3.53	n.i. n.i.
16.6	**CE-2**	489.1019	979.2121	491.1181			C_23_H_22_O_12_	2.86, 2.35, 1.83	n.i.
17.2	**CE-2**	475.1588 n.o.	951.3253 n.o.	n.o. 494.2024	n.o. n.o.	n.o. n.o.	C_24_H_28_O_10_ C_24_H_32_NO_10_	3.36, 3.57 0.40	n.i. n.i.
22.5	**CE-1**	343.0805	n. o.	345.0964	n.o.	711.1695	C_18_H_16_O_7_	3.79, 2.89, 0.70	Penduletin*
22.9	**CE-1**	373.0908	747.1892	375.1080	n.o.	771.1897	C_19_H_18_O_8_	4.02, 4.42, 0.00, 0.52	Casticin*
23.4	**CE-1**	283.0598	n. o.	285.0753	n.o.	n.o.	C_16_H_12_O_5_	2.83, 3.51	n.i.
23.7	**CE-2**	313.0711	n. o.	315.0864	n.o.	651.1473	C_17_H_14_O_6_	0.32, 1.58, 0.77	n.i.
27.9	**CE-1**	n. o.	n. o.	359.1132	n.o.	739.2017	C_19_H_18_O_7_	0.28, −1.89	Penduletin-4′-methyl ether*

CE-1, crude ethanol S. perforatum extract from harvest 1; CE-2, crude ethanol S. perforatum extract from harvest 2; n.o., not observed; n.i., not identified. * confirmed by isolation and NMR data.

### 3.3 Validation and quantitative analyses

**Figure 3:**
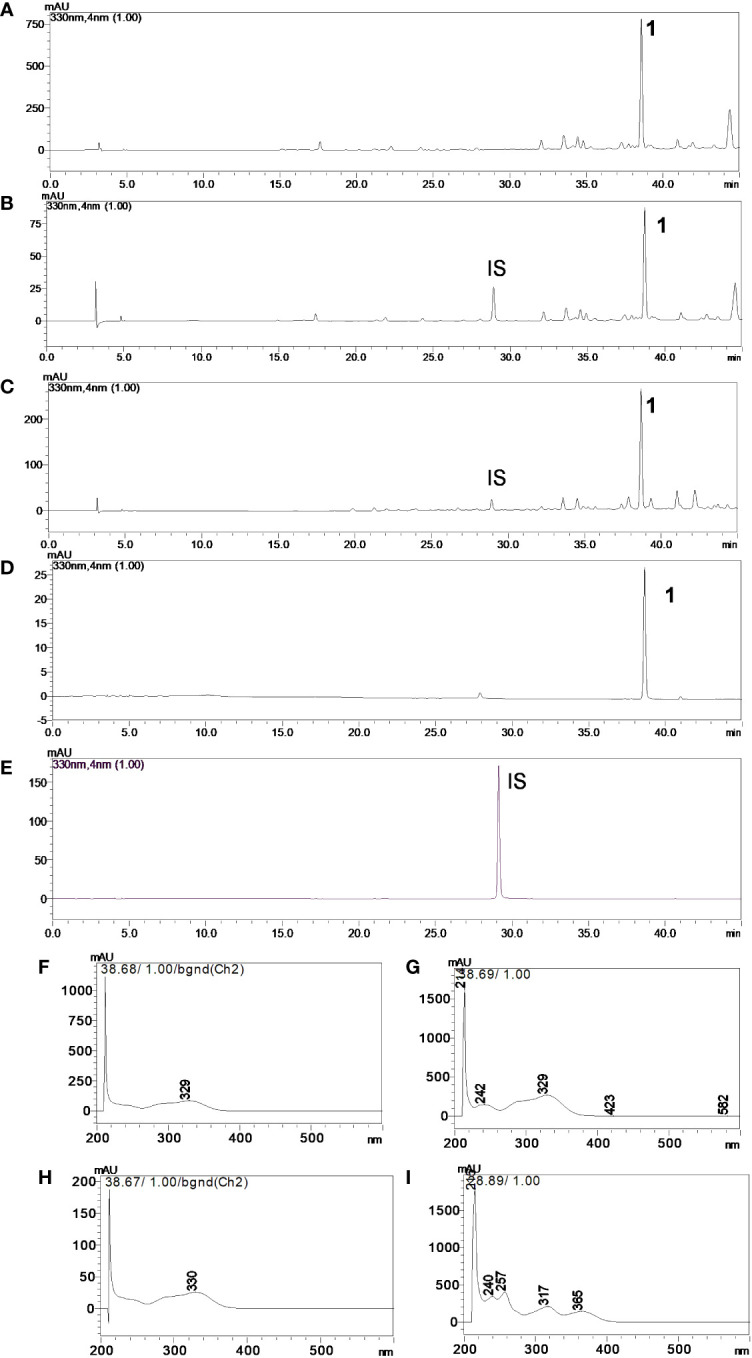
Chromatograms in the UV region, λ = 330 nm of **(A)** crude ethanol *Stizophyllum perforatum* leaf extract (CE-2), **(B)** crude ethanol *S. perforatum* leaf extract (CE-2) and IS, **(C)** crude ethanol *S. perforatum* leaf extract (CE-1) and IS, **(D)** verbascoside (**1**), and **(E)** mangiferin (**IS**). UV-Vis spectra of **(F)** verbascoside (**1**) in CE-2, **(G)** verbascoside (**1**) in CE-1, **(H)** verbascoside (**1**), and **(I)** mangiferin (**IS**). Analytical conditions: acetonitrile/water (+0.1% acetic acid) linear gradient from 2% to 25% acetonitrile for 40 min, 25% to 100% acetonitrile in 5 min, and 100% acetonitrile for 5 min. The flow rate was 1.0 mL/min, and the ODS column was Phenomenex Luna.

The chromatographic condition (**Figures 3A–E**; linear gradient of solvent A (2% aqueous acetic acid) and solvent B (acetonitrile)) was as follows: 2%–25% B over 40 min, followed by 25%–100% B over 5 min and 100% B for 5 min) allowed **1** (R_t_ of 38.68 min) to be separated from the other compounds in CE-1 and CE-2; mangiferin (R_t_ of 28.89 min) was the **IS**. We also obtained the UV-Vis spectra (**Figures 3F–I**) in this condition. Peak purity, obtained with the aid of the DAD detector, was 0.999 for both **1** and **IS**, confirming that no coelution occurred. We investigated linearity using external and internal standardization methods. At 330 nm, the method was linear for **1** at concentrations between 0.8 and 60.0 µg/mL. The regression equation was y = 34,237x − 23,016, the correlation coefficient (r) was 0.9920, and the RSD was less than 5% for triplicate analyses. At 330 nm, the **IS** analytical curve was also linear in the same concentration range. The regression equation was y = 24,680x − 31,022, r was 0.9948, and RSD was less than 5% for triplicate analyses. As for the internal standardization calibration curve, the equation was y = 0.1685x − 0.1157, with r = 0.9958 and RSD < 5%. The LOD and LOQ were 0.30 and 1.01 µg/mL, 0.05 and 0.17 µg/mL, and 0.36 and 1.19 µg/mL, respectively. **IS** recovery from the CE-2 solutions spiked with **IS** at 3.0, 10.0, and 50.0 µg/mL was 102.82%, 108.51%, and 111.04%, respectively, with RSD < 5%. Regarding repeatability, RSD was 0.64%, 1.48%, and 1.76%, respectively. With respect to intermediate precision, RSD was 3.15%, 0.60%, and 1.50%, respectively. We determined that the concentration of **1** in CE-1 and CE-2 was 3.51 ± 0.20 and 3.78 ± 0.14 µg/mL, or 0.59% and 0.63% relative to the dry crude ethanol *S. perforatum* leaf extract, respectively.

### 3.4 Acute toxicity

In this study, animals treated with 2,000 mg of CE-2/kg of body weight for 14 days did not die or have significantly altered behavior. All the animals presented an average weight gain of 31.00 ± 2.35 g in this period. The macroscopic pathological necropsy did not show alterations that could indicate pathologies in organs and tissues. The histopathological analysis did not indicate alterations typical of pathologies in the kidney and liver (**Table 2**) . On the basis of the GHS classification for acute toxicity of chemicals, we classified CE-2 in category 5 (LD_50_ > 2,000–5,000 mg/kg of body weight).

**Table 2:** Histopathological analysis data of liver and kidney treated with crude ethanol *Stizophyllum perforatum* leaf extract.

Slides	Necrosis	Angiogenesis	PMII	MII	Fibroplasia	Congestion	Degeneration	Megalocytosis
**51-6-K**	0 ± 0	0 ± 0	0 ± 0	0 ± 0	0 ± 0	1 ± 1	0 ± 0	0 ± 0
**51-6-L**	0 ± 0	0 ± 0	0 ± 0	1 ± 0	0 ± 0	1 ± 0	1 ± 0	1 ± 0
**Score**	0 (absent)	1 (mild)	2 (moderated)	3 (intense)				

Data represent mean ± SD.

PMII, polymorphonuclear inflammatory infiltrate; MII, mononuclear inflammatory infiltrate; K, kidney; L, liver.

### 3.5 Antileishmanial and cytotoxic activities

We evaluated the antileishmanial activity of the samples, namely, CE-1, CE-2, HF, EAF, HMF, EAF-1, EAF-2, EAF-3, EAF-4, **1**, and **3**, against *L. amazonensis* promastigotes and amastigotes (**Table 3**). First, we screened the samples at 50 µg/mL or µM against *L. amazonensis* promastigotes to find that only CE-1, CE-2, HF, EAF, EAF-3, **1**, and **3** displayed over 70% antileishmanial activity after 24 h. CE-1, CE-2, EAF, and EAF-3 showed EC_50_ lower than 10 µg/mL after 24 h, whereas **1** and **3** presented EC_50_ of 3.89 and 9.06 µg/mL (6.23 and 24.20 µM), respectively, so **3** was 3.9-fold less active than **1**. Furthermore, CE-1 and **1** provided similar EC_50_ against *L. amazonensis* promastigotes.

**Table 3:** Antileishmanial and cytotoxic activities of crude ethanol *Stizophyllum perforatum* leaf extracts and isolated compounds.

	Leishmania amazonensis					Cytotoxicity
Samples	Promastigote			Intracellular amastigote		
	[50 µg/mL or 50 µM]^a^	EC_50_ ^b^ µg/mL	95% CI^c^	EC_50_ ^b^ µg/mL	95% CI^c^	CC_50_ ^d^ µg/mL or µM
CE-1	86.39 ± 1.12	4.44	2.82–6.06	15.57	8.53–28.42	>100
CE-2	82.08 ± 5.10	7.30	5.38–9.15	8.21	3.15–21.33	>100
HF	75.66 ± 7.57	36.23	27.65–47.46	>50	–	>100
EAF	92.33 ± 3.78	5.66	1.13–10.15	>50	–	>100
HMF	43.00 ± 6.00	–	–	>50	–	>100
EAF-1	55.99 ± 5.07	–	–	>50	–	>100
EAF-2	28.57 ± 6.30	–	–	>50	–	>100
EAF-3	90.05 ± 1.97	5.80	2.15–9.35	16.53	8.67–31.52	>100
EAF-4	59.47 ± 4.07	–	–	>50	–	>100
**Casticin**	78.65 ± 3.15	9.06 (24.20 µM)	18.27–30.12	7.10 (18.97 µM)	17.25–20.86	>100
**Verbascoside**	80.72 ± 2.18	3.89 (6.23 µM)	4.36–8.91	2.32 (3.71 µM)	0.96–14.51	>100
**Amphotericin B**	–	0.014 (0.011 µM)	0.009–0.023	0.086 (0.093 µM)	0.079–0.11	42.9 (46.3 µM)

CE-1, crude ethanol S. perforatum leaf extract from harvest 1; CE-2, crude ethanol S. perforatum leaf extract from harvest 2; HF, n-hexane fraction; EAF, ethyl acetate fraction; HMF, hydromethanol fraction; EAF-1, subfraction 1 from EAF; EAF-2, subfraction 2 from EAF; EAF-3, subfraction 3 from EAF; EAF-4, subfraction 4 from EAF; -, not calculated.

a Screening, % of promastigote inhibition ± SD (24 h).

b EC_50_ 50% inhibitory concentration of parasites [µg/mL or µM].

c Confidence interval. Data of two and/or three independent experiments.

d CC_50_: half-maximal cytotoxic concentration for 50% of cells.

CE-1 (EC_50 =_ 15.57 µg/mL), CE-2 (EC_50 =_ 8.21 µg/mL), EAF-3 (EC_50 =_ 16.53 µg/mL), **1** (EC_50 =_ 2.32 µg/mL or 3.71 µM), and **3** (EC_50 =_ 7.10 µg/mL or 18.97 µM) exerted antileishmanial effects on *L. amazonensis* amastigotes. **1** afforded the most promising EC_50_ against *L. amazonensis* amastigotes and was 5.1-, 6.7-, 3.5-, and 7.1-fold more effective than **3**, CE-1, CE-2, and EAF, respectively. In turn, **3** was more effective against *L. amazonensis* amastigotes than CE-1 and EAF-3.

**Figure 4:**
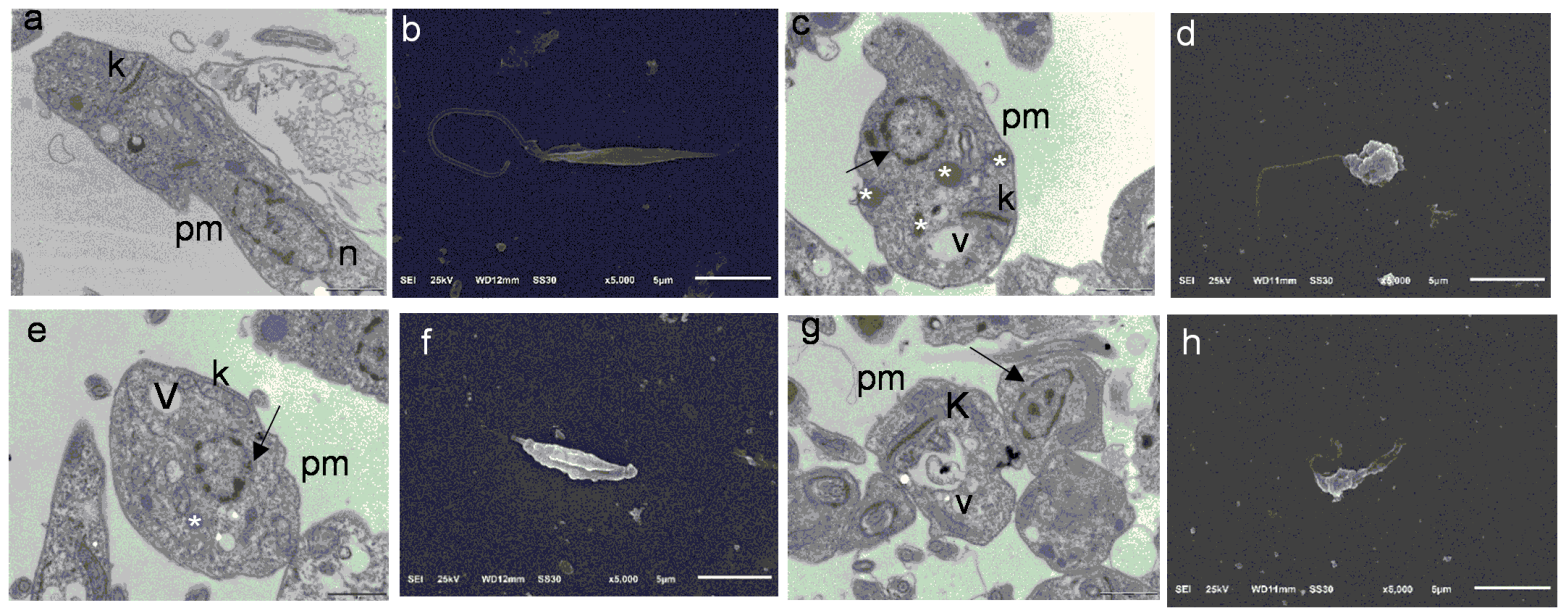
Transmission electron microscopy and scanning electron microscopy images of *Leishmania amazonensis* promastigotes (24 h). **(A)** Negative control (RPMI 1640 and 0.1% dimethyl sulfoxide (DMSO)), bars = 1 µm. **(B)** Negative control (RPMI 1640 and 0.1% DMSO), bars = 5 µm. **(C)** Amphotericin B, bars = 1 µm. **(D)** Amph. B, bars = 5 µm. **(E)** Verbascoside (IC_50_ 8.92 µM), bars = 1 µm. **(F)** Verbascoside (IC_50_ 8.92 µM), bars = 5 µm. **(G)** Verbascoside (IC_90_ 89.7 µM), bars = 1 µm. **(H)** Verbascoside (IC_90_ 89.7 µM), bars = 5 µm. Kinetoplast (k), lipid accumulation (white asterisk), nucleus (n), nucleus disorganization (arrow), plasma membrane (pm), and vacuole (v).

To assess whether **1** promoted morphological and structural changes in *L. amazonensis* promastigotes, we performed SEM and TEM analyses of *L. amazonensis* promastigotes incubated with **1** at 8.92 µM (EC_50_) or 89.7 µM (EC_90_) for 24 h. SEM analyses (**Figure 4**) showed morphological changes in *L. amazonensis* promastigotes incubated with amphotericin B (positive control, **Figure 4D**) or **1** (**Figures 4F, H**) when compared to the negative control (0.1% dimethyl sulfoxide, **Figure 4B**). In the negative control group, *L. amazonensis* promastigotes presented an elongated shape (**Figure 4B**). Compared to the negative control, *L. amazonensis* promastigotes incubated with **1** (**Figures 4F, H**) underwent changes such as a reduced number of parasites, acquisition of a rounded shape, and altered plasma membrane. TEM revealed an elongated body and intact organelles (nucleus, plasma membrane, and kinetoplast) in the negative control (**Figure 4A**). *L. amazonensis* promastigotes incubated with amphotericin B and positive control (**Figure 4C**) presented vacuolization of the cytoplasm, swollen kinetoplasts, lipid bodies, and altered nuclear membrane. As for *L. amazonensis* promastigotes incubated with **1** at 8.92 µM (**Figure 4E**) or 89.7 µM (**Figure 4G**), they displayed vacuolization of the cytoplasm, lipid bodies, altered nuclear membrane, interstitial extravasation, and swollen kinetoplasts.

We evaluated the cytotoxic activity of CE-1, CE-2, HF, EAF, EAF-3, **1**, and **3** against the murine macrophage cell line (J774A.1). None of the samples were cytotoxic under the evaluated conditions: CC_50_ was greater than 100 µg/mL or µM.

## 4 Discussion

The phytochemical study of the crude ethanol leaf extract obtained from *S. perforatum* indicated that this species biosynthesizes triterpenes, phenylethanoid glycosides, and flavonoids. Among these compounds, only **5** had previously been isolated from *S. riparium*, a morphologically similar species (Duh et al., 1987; Beyer et al., 2017). To the best of our knowledge, this is the first report of **1**–**4** and **6**–**8** in *Stizophyllum*.

Chromatographic fingerprinting of CE-1 and CE-2 showed some differences in their chemical composition, well established and influenced by factors such as plant source, growth, harvest, season, and water availability (Chaves et al., 1997; Copaja et al., 2003; Soni et al., 2015; Gobbo-Neto et al., 2017; Prinsloo and Nogemane, 2018; Sampaio and Da Costa, 2018; Gimenez et al., 2020). However, we also observed some similarities, including the peak at R_t_ of 11.7 min, which revealed that **1** is present in both CE-1 and CE-2. Thus, we selected **1** as the chemical marker for *S. perforatum*. Interestingly, **1** has also been described in other Bignoniaceae species, such as *Cuspidaria pulchra* and *Xylophragma harleyi* (Lima et al., 2003; Alvarenga et al., 2015). The phytochemical study of *S. riparium* resulted in the isolation of triterpene and pregnane derivatives (Duh et al., 1987). Additionally, we compared the formulas obtained from the HPLC–HRMS characterization with the formulas of previously described compounds, but we were unable to identify any of them. Nevertheless, we were able to identify **5** by NMR data after it was isolated.

The developed HPLC method is suitable for controlling the quality of herbal materials from *S. perforatum*, and according to Indrayanto (2022), method validation in plant science is crucial for developing modern drugs from herbal medicines.

Our results concerning the antipromastigote and antiamastigote activities of CE-1 and CE-2 are more promising than the results reported by Maquiaveli et al. (2016b), who isolated **1** and isoverbascoside from the *n*-butanol fraction of *Stachytarpheta cayennensis*, tested them against *L. amazonensis* promastigotes and amastigotes, and obtained EC_50_ of 51 and 32 µg/mL, respectively. In addition to that, a previous study reported that the crude leaf extract obtained from *S. perforatum* exhibits trypanocidal activity with an EC_50_ of 20.2 µg/mL (Silva et al., 2014). Another study, which also isolated **5** from *Ajuga laxmannii*, showed that the crude methanol extract of this plant has EC_50_ of 30.1 µg/mL against *Leishmania donovani* axenic amastigotes (Atay et al., 2016). Here, we determined that the concentration of **1** in CE-1 and CE-2 is 3.51 ± 0.20 and 3.78 ± 0.14 µg/mL, or 0.59% and 0.63% relative to the dry crude ethanol leaf extract, respectively. However, the concentrations of **1** in the plant extracts mentioned in the literature were not established.

Verbascoside (**1**) has various biological activities, including antioxidant, anti-inflammatory, antineoplastic, wound-healing, neuroprotective, and antileishmanial activity properties (Alipieva et al., 2014; Atay et al., 2016; Maquiaveli et al., 2016a; Maquiaveli et al., 2017). **1** has an EC_50_ of 19 μM against *L. amazonensis* promastigotes and is a competitive arginase inhibitor (Ki = 0.7 μM) (Maquiaveli et al., 2016a). Additionally, **1** inhibits *L. amazonensis* amastigotes (EC_50 =_ 32 μM) by selectively inhibiting arginase, which reduces the protective oxidative mechanism of the parasite and impairs trypanothione synthesis. Arginase belongs to the polyamine biosynthesis pathway, which is important for parasite infectivity (Maquiaveli et al., 2017). Our results are more promising—**1** has EC_50_ of 6.23 and 3.71 μM for *L. amazonensis* promastigotes and amastigotes, respectively. The antileishmanial activity of **1** has also been established against *L. donovani* axenic amastigotes (8.7 µg/mL) (Kirmizibekmez et al., 2004; Atay et al., 2016).

Flavonols exhibit antileishmanial activity against *L. donovani* axenic amastigotes (Tasdemir et al., 2006; Singh et al., 2014). Quercetin has been evaluated against *L. amazonensis-*infected macrophages, and its IC_50_ is 3.4 μM (Fonseca-Silva et al., 2013). Regarding flavonols, quercetin is a mixed inhibitor of *L. amazonensis* arginase, whereas quercitrin and isoquercitrin are uncompetitive inhibitors of this enzyme (da Silva et al., 2012).

Triterpenes, such as **5** and **6**, isolated from the bioactive fractions, also present antileishmanial activity against *L. amazonensis* promastigotes, *Leishmania major* promastigotes, and intracellular *L. amazonensis* amastigotes to some extent (Torres-Santos et al., 2004; Peixoto et al., 2011; Begum et al., 2014; Yamamoto et al., 2015). According to Yamamoto et al. (2015), **5** presents IC_50_ of 6.2 ± 1.8 µg/mL against promastigotes, **6** is inactive, and **5** alters the ultrastructure in *L. amazonensis* promastigotes.

The presence of these compounds in CE-1 and CE-2 can contribute to antileishmanial activity. These findings are often considered to result from a synergistic or additive effect of the constituents (Caesar and Cech, 2019). Our results provide evidence of the antileishmanial activity induced by *S. perforatum* and additional evidence of the antileishmanial activity of **1** and **3**. Further *in vivo* studies should be conducted to assess the antileishmanial activity of the *S. perforatum* crude extract against *Leishmania*.

## 5 Conclusion

We isolated verbascoside (**1**), penduletin (**2**), casticin (**3**), penduletin 4′-methyl ether (**4**), ursolic acid (**5**), oleanolic acid (**6**), stigmasterol (**7**), and β-sitosterol (**8**) from CE-1 using chromatographic techniques and identified these compounds by NMR spectrometry.

HPLC–HRMS data showed that **1** is present in both CE-1 and CE-2, confirming that the extracts exhibit similar but not identical chemical composition.

The evaluated samples, CE-1, CE-2, EAF, EAF-3, **1**, and **3**, display antileishmanial activity against *L. amazonensis* promastigotes and amastigotes. The antiparasitic effect of **1** was confirmed by ultrastructural changes in the parasite. Nevertheless, the samples showed low cytotoxicity in acute oral tests. Thus, *S. perforatum* is a suitable candidate for further *in vivo* investigations against *L. amazonensis*.

## Data availability statement

The original contributions presented in the study are included in the article/**Supplementary Material**. Further inquiries can be directed to the corresponding author.

## Ethics statement

The animal study was approved by Ethics Committee on the Use of Animals at the University of Franca (CEUA) number 8994060220. The study was conducted in accordance with the local legislation and institutional requirements.

## Author contributions

OA: Conceptualization, Writing – original draft, Data curation, Methodology, Formal analysis. SO: Data curation, Investigation, Writing – original draft. LM: Formal analysis, Writing – review & editing. AC: Writing – review & editing, Formal analysis, Methodology. VG: Formal analysis, Investigation, Writing – review & editing. MS: Resources, Writing – review & editing. WC: Resources, Writing – review & editing. AJ: Resources, Writing – review & editing. DT: Conceptualization, Funding acquisition, Resources, Writing – original draft. LM: Conceptualization, Funding acquisition, Resources, Writing – review & editing. PP: Conceptualization, Funding acquisition, Resources, Supervision, Writing – original draft, Writing – review & editing.

## References

[B1] Alipieva K. Korkina L. Orhan I. E. Georgiev M. I. (2014). Verbascoside: a review of its occurrence, (bio)synthesis and pharmacological significance. Biotechnol. Adv. 32, 1065–1076. doi: 10.1016/j.bioteChadv.2014.07.001 25048704

[B4] Alvarenga T. A. Alves O. A. Pagotti M. C. Cunha W. R. Silva M. L. A. E. Sales J. D.. (2021). *In vitro* antileishmanial activity of *Anacardium othonianum* and isolated compounds against *Leishmania amazonensis* . Acta Brasiliensis 5, 44–47. doi: 10.22571/2526-4338429

[B2] Alvarenga T. A. Bertanha C. S. de Oliveira P. F. Tavares D. C. Gimenez V. M. Silva M. L.. (2015). Lipoxygenase inhibitory activity of *Cuspidaria pulchra* and isolated compounds. Nat. Prod. Res. 29, 1083–1086. doi: 10.1080/14786419.2014.981182 25428032

[B3] Alvarenga T. A. de Oliveira P. F. de Souza J. M. Tavares D. C. Andrade E Silva M. L. Cunha W. R.. (2016). Schistosomicidal Activity of Alkyl-phenols from the Cashew *Anacardium occidentale* against *Schistosoma mansoni* Adult Worms. J. Agric. Food Chem. 64, 8821–8827. doi: 10.1021/acs.jafc.6b04200 27934289

[B5] ANVISA (2017). Critérios para a validação de métodos analíticos, Resolução RDC n° 166. Agência Nacional de Vigilância Sanitária (Brasília: Diário Oficial da República Federativa do Brasil, Imprensa Nacional).

[B6] Atay I. Kirmizibekmez H. Kaiser M. Akaydin G. Yesilada E. Tasdemir D. (2016). Evaluation of in *vitro* antiprotozoal activity of *Ajuga laxmannii* and its secondary metabolites. Pharm. Biol. 54, 1808–1814. doi: 10.3109/13880209.2015.1129542 26734766

[B7] Begum S. Ayub A. Qamar Zehra S. Shaheen Siddiqui B. Iqbal Choudhary M. (2014). Leishmanicidal triterpenes from *Lantana camara* . Chem. Biodivers. 11, 709–718. doi: 10.1002/cbdv.201300151 24827681

[B8] Bertanha C. S. Gimenez V. M. M. Furtado R. A. Tavares D. C. Cunha W. R. Silva M. L. A.. (2020). Isolation, in *vitro* and in *silico* Evaluation of Phenylethanoid Glycoside from *Arrabidaea brachypoda* as Lipoxygenase Inhibitor. J. Braz. Chem. Soc 31, 849–855. doi: 10.21577/0103-5053.20190248

[B9] Beyer M. Nazareno A. G. Lohmann L. G. (2017). Using genomic data to develop chloroplast DNA SSRs for the Neotropical liana *Stizophyllum riparium* (Bignonieae, Bignoniaceae). Appl. Plant Sci. 5, apps.1700061. doi: 10.3732/apps.1700061 29109920 PMC5664965

[B10] Brandão G. C. Kroon E. G. Souza D. E. R. Souza Filho J. D. Oliveira A. B. (2013). Chemistry and antiviral activity of *Arrabidaea pulchra* (Bignoniaceae). Molecules 18, 9919–9932. doi: 10.3390/molecules18089919 23959197 PMC6269977

[B11] Caesar L. K. Cech N. B. (2019). Synergy and antagonism in natural product extracts: when 1 + 1 does not equal 2. Nat. Prod. Rep. 36, 869–888. doi: 10.1039/c9np00011a 31187844 PMC6820002

[B12] Chaves N. Escudero J. C. Gutierrez-Merino C. G. (1997). Role of ecological variables in the seasonal variation of flavonoids contente of *Citrus ladanifer* exudate. J. Chem. Ecol. 23, 579–603. doi: 10.1023/B:JOEC.0000006398.79306.09

[B13] Citoglu G. S. Sever B. Antus S. Baitz-Gacs E. Altanlar N. A. F. Citoglu G. S.. (2004). Antifungal diterpenoids and flavonoids from *Ballota inaequidens* . Pharm. Biol. 42, 659–663. doi: 10.1080/13880200490902626

[B14] Copaja S. V. Blackburn C. Carmona R. (2003). Variation of saponin contents in *Quillaja saponaria* Molina. Wood Sci. Technol. 37, 103–108. doi: 10.1007/s00226-002-0150-8

[B15] da Silva E. R. Maquiaveli C. do C. Magalhães P. P. (2012). The leishmanicidal flavonols quercetin and quercitrin target *Leishmania* (Leishmania) *amazonensis* arginase. Exp. Parasitol. 130, 183–188. doi: 10.1016/j.exppara.2012.01.015 22327179

[B17] Duh C. Y. Kinghorn A. D. Pezzuto J. M. (1991). Cell-cycle specific cytotoxicity mediated by stizophyllin (2 alpha,3 beta,12 beta-trihydroxypregna-4,7,16-trien-20-one), a novel electrophilic pregnane isolated from *Stizophyllum riparium* . Chem. Biol. Interact. 80, 43–56. doi: 10.1016/0009-2797(91)90030-b 1913978

[B16] Duh C. Y. Pezzuto J. M. Kinghorn A. D. Leung S. L. Farnsworth N. R. (1987). Plant anticancer agents XLIV. Cytotoxic constituents from *Stizophyllum riparium* . J. Nat. Prod. 50, 63–74. doi: 10.1021/np50049a010 3598599

[B18] Fonseca-Silva F. Inacio J. D. Canto-Cavalheiro M. M. Almeida-Amaral E. E. (2013). Reactive oxygen species production by quercetin causes the death of *Leishmania amazonensis* intracellular amastigotes. J. Nat. Prod. 76, 1505–1508. doi: 10.1021/np400193m 23876028

[B19] Gimenez V. M. M. e Silva M. L. A. Cunha W. R. Januario A. H. Costa E. J. X. Pauletti P. M. (2020). Influence of environmental, geographic, and seasonal variations in the chemical composition of *Miconia* species from Cerrado. Biochem. Syst. Ecol. 91, 104049. doi: 10.1016/j.bse.2020.104049

[B20] Gobbo-Neto L. Bauermeister A. Sakamoto H. T. Gouvea D. R. Lopes J. L. C. Lopes N. P. (2017). Spatial and temporal variations in secondary metabolites content of the Brazilian arnica leaves (*Lychnophora ericoides* mart., asteraceae). J. Braz. Chem. Soc 28, 2382–2390. doi: 10.21577/0103-5053.20170092

[B21] Goulart M. O. F. Sant’Ana A. E. G. Lima R. A. Cavalcante S. H. Carvalho M. G. Braz-Filho R. B. (1993). Fitoconstituintes químicos isolados de *Jatropha elliptica*, atribuição dos deslocamentos químicos dos átomos de carbono e hidrogênio dos diterpenos jatrofolonas a e b. Quim. Nova. 16, 95–100. Available at: https://s3.sa-east-1.amazonaws.com/static.sites.sbq.org.br/quimicanova.sbq.org.br/pdf/Vol16No2_95_v16_n2_%283%29.pdf.

[B22] Gupta M. P. Correa M. D. Solís P. N. Jones A. Galdames C. Guionneau-Sinclair F. (1993). Medicinal plant inventory of Kuna Indians: Part 1. J. Ethnopharmacol. 40, 77–109. doi: 10.1016/0378-8741(93)90054-9 8133656

[B23] Indrayanto G. (2022). The importance of method validation in herbal drug research. J. Pharm. Biomed. Anal. 214, 114735. doi: 10.1016/j.jpba.2022.114735 35344789

[B24] Kanchanapoom T. Kasai R. Yamasaki K. (2002). Phenolic glycosides from *Barnettia kerrii* . Phytochemistry 59, 565–570. doi: 10.1016/s0031-9422(01)00476-9 11853753

[B25] Kirmizibekmez H. Calis I. Perozzo R. Brun R. Dönmez A. A. Linden A.. (2004). Inhibiting activities of the secondary metabolites of *Phlomis brunneogaleata* against parasitic protozoa and plasmodial enoyl-ACP Reductase, a crucial enzyme in fatty acid biosynthesis. Planta Med. 70, 711–717. doi: 10.1055/s-2004-827200 15326547

[B26] Lima A. S. A. Amorim E. L. C. Sena K. X. F. R. Chiappeta A. A. Nunes X. P. Agra M. F.. (2003). Antimicrobial activity of a mixture of two isomeric phenylpropanoid glycosides from *Arrabidaea harleyi* A.H. Gentry (Bignoniaceae). Rev. Bras. Cienc. Farm. 39, 77–81. doi: 10.1590/S1516-93322003000100008

[B27] Lohmann L. G. Taylor C. M. (2014). A new generic classification of tribe Bignonieae (Bignoniaceae). Ann. Mo. Bot. Gard. 99, 348–489. doi: 10.3417/2003187

[B28] Maquiaveli C. C. Lucon-Júnior J. F. Brogi S. Campiani G. Gemma S. Vieira P. C.. (2016a). Verbascoside inhibits promastigote growth and arginase activity of *Leishmania amazonensis* . J. Nat. Prod. 79, 1459–1463. doi: 10.1021/acs.jnatprod.5b00875 27096224

[B29] Maquiaveli C. D. C. Oliveira E. Sá A. M. Vieira P. C. da Silva E. R. (2016b). *Stachytarpheta cayennensis* extract inhibits promastigote and amastigote growth in *Leishmania amazonensis* via parasite arginase inhibition. J. Ethnopharmacol. 192, 108–113. doi: 10.1016/j.jep.2016.07.044 27432217

[B30] Maquiaveli C. D. C. Rochetti A. L. Fukumasu H. Vieira P. C. da Silva E. R. (2017). Antileishmanial activity of verbascoside: Selective arginase inhibition of intracellular amastigotes of *Leishmania* (*Leishmania*) *amazonensis* with resistance induced by LPS plus IFN-gamma. Biochem. Pharmacol. 127, 28–33. doi: 10.1016/j.bcp.2016.12.018 28017773

[B31] OECD (2022). Test No. 425: Acute Oral Toxicity: Up-and-Down Procedure, OECD Guidelines for the Testing of Chemicals, Section 4 (Paris: OECD Publishing). doi: 10.1787/9789264071049-en

[B32] Oliveira T. A. S. Vieira T. M. Esperandim V. R. Martins C. H. G. Magalhães L. G. Miranda M. L. D.. (2022). Antibacterial, antiparasitic, and cytotoxic activities of chemical characterized essential oil of *Chrysopogon zizanioides* roots. Pharm. (Basel) 15, 967. doi: 10.3390/ph15080967 PMC9415812 36015115

[B33] Pauletti P. M. Castro-Gamboa I. Siqueira Silva D. H. Young M. C. Tomazela D. M. Eberlin M. N.. (2003). New antioxidant *C*-glucosylxanthones from the stems of *Arrabidaea samydoides* . J. Nat. Prod. 66, 1384–1387. doi: 10.1021/np030100t 14575443

[B34] Peixoto J. A. Andrade E Silva M. L. Crotti A. E. Cassio Sola Veneziani R. Gimenez V. M. Januário A. H.. (2011). Antileishmanial activity of the hydroalcoholic extract of *Miconia langsdorffii*, isolated compounds, and semi-synthetic derivatives. Molecules 16, 1825–1833. doi: 10.3390/molecules16021825 21343887 PMC6259650

[B35] Pereira A. C. Silva M. L. Souza J. M. Laurentiz R. S. Rodrigues V. Januário A. H.. (2015). *In vitro* and in *vivo* anthelmintic activity of (-)-6,6’-dinitrohinokinin against schistosomula and juvenile and adult worms of *Schistosoma mansoni* . Acta Trop. 149, 195–201. doi: 10.1016/j.actatropica.2015.06.005 26071648

[B36] Prinsloo G. Nogemane N. (2018). The effects of season and water availability on chemical composition, secondary metabolites and biological activity in plants. Phytochem. Rev. 17, 889–902. doi: 10.1007/s11101-018-9567-z

[B37] Sampaio B. L. Da Costa F. B. (2018). Influence of abiotic environmental factors on the main constituents of the volatile oils of *Tithonia diversifolia* . Rev. Bras. Farmacogn. 28, 135–144. doi: 10.1016/j.bjp.2018.02.005

[B38] Sevindik H. G. Ozgen U. Atila A. Ozturk, Er. H. Kazaz C. Duman H. (2015). Phtytochemical Studies and Quantitative HPLC Analysis of Rosmarinic Acid and Luteolin 5-O-β-D-Glucopyranoside on *Thymus praecox* subsp. grossheimii var. grossheimii. Chem. Pharm. Bull. 63, 720–725. doi: 10.1248/cpb.c14-00877 26329865

[B39] Silva F. A. J. Nakaima Kohatsu A. A. Regasini L. O. Marx Young M. C. Bolzani V. S. Siqueira Silva D. H.. (2014). Trypanocidal activity evaluation of *Distictella mansoana* (DC.) Bureau and *Stizophyllum perforatum* (Cham.) Miers extracts. Planta Med. 80, P2B106. doi: 10.1055/s-0034-1394983

[B5000] Singh N. Mishra B. B. Bajpai S. Singh R. K. Tiwari V. K.. (2014). Natural product based leads to fight against leishmaniasis. Bioorg. Med. Chem. 22, 18-45. doi: 10.1016/j.bmc.2013.11.048 24355247

[B40] Soni U. Brar S. Gauttam V. K. (2015). Effect of seasonal variation on secondary metabolites of medicinal plants. Int. J. Pharm. Sci. Res. 6, 3654–3662. doi: 10.13040/IJPSR.0975-8232.6(9).3654-62

[B41] Tasdemir D. Kaiser M. Brun R. Yardley V. Schmidt T. J. Tosun F.. (2006). Antitrypanosomal and antileishmanial activities of flavonoids and their analogues: in *vitro*, in *vivo*, structure-activity relationship, and quantitative structure-activity relationship studies. Antimicrob. Agents Chemother. 50, 1352–1364. doi: 10.1128/AAC.50.4.1352-1364.2006 16569852 PMC1426963

[B42] Torres-Santos E. C. Lopes D. Oliveira R. R. Carauta J. P. Falcao C. A. Kaplan M. A.. (2004). Antileishmanial activity of isolated triterpenoids from *Pourouma guianensis* . Phytomedicine 11, 114–120. doi: 10.1078/0944-7113-00381 15070160

[B43] Valadeau C. Castillo J. A. Sauvain M. Lores A. F. Bourdy G. (2010). The rainbow hurts my skin: medicinal concepts and plants uses among the Yanesha (Amuesha), an Amazonian Peruvian ethnic group. J. Ethnopharmacol. 127, 175–192. doi: 10.1016/j.jep.2009.09.024 19835943

[B44] Valerino-Díaz A. B. Zanatta A. C. Gamiotea-Turro D. Candido A. C. B. B. Magalhães L. G. Vilegas W.. (2020). An enquiry into antileishmanial activity and quantitative analysis of polyhydroxylated steroidal saponins from *Solanum paniculatum* L. leaves. J. Pharm. Biomed. Anal. 191, 113635. doi: 10.1016/j.jpba.2020.113635 32998105

[B45] Wang Y. Hamburger M. Gueho J. Hostettmann K. (1989). Antimicrobial flavonoids from *Psiadia trinervia* and their methylated and acetylated derivatives. Phytochemistry 28, 2323–2327. doi: 10.1016/S0031-9422(00)97976-7

[B46] Yamamoto E. S. Campos B. L. Jesus J. A. Laurenti M. D. Ribeiro S. P. Kallás E. G.. (2015). The effect of ursolic acid on leishmania (*Leishmania*) *amazonensis* is related to programed cell death and presents therapeutic potential in experimental cutaneous leishmaniasis. PloS One 10, e0144946. doi: 10.1371/journal.pone.0144946 26674781 PMC4699202

