# Supplementary material for: HPLC method for quantifying verbascoside in Stizophyllum perforatum and assessment of verbascoside acute toxicity and antileishmanial activity
Source: Front Plant Sci. 2023 Dec 8;14:1324680. doi: 10.3389/fpls.2023.1324680 (PMC10749199; doi:10.3389/fpls.2023.1324680)
Supplement: Supplementary file 1 [file DataSheet_1.docx]

HPLC method for the quantification of verbascoside in *Stizophyllum perforatum*, acute toxicity and antileishmanial activity.

Osvaine Junior Alvarenga Alves^1^, Saulo Duarte Ozelin^1^, Larissa F. Magalhães^1^, Ana Carolina Bolela Bovo Candido^1^, Valéria Maria Melleiro Gimenez ^1,2^, Márcio Luís Andrade e Silva^1^, Wilson Roberto Cunha^1^, Ana Helena Januario^1^, Denise Crispim Tavares^1^, Lizandra Guidi Magalhães^1^ and Patricia Mendonça Pauletti^1*^

^1^ Center for Research in Exact and Technological Sciences, University of Franca, Franca, São Paulo, Brazil

^2^ Faculty of Animal Science and Food Engineering, University of São Paulo, Pirassununga, São Paulo, Brazil

*** Correspondence:**Patricia Mendonça Pauletti
patricia.pauletti@unifran.edu.br

Supplementary Material

**
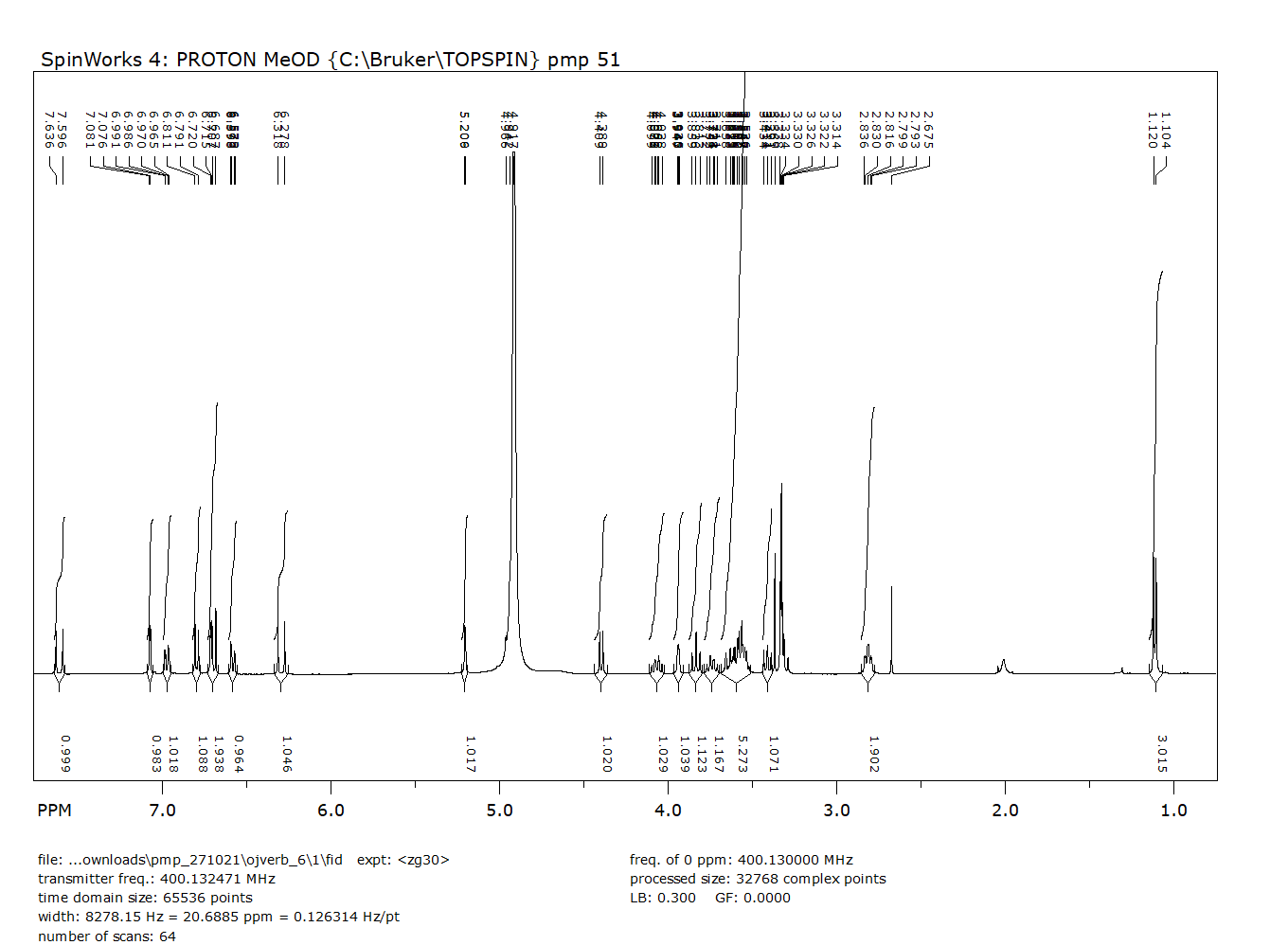
**

**Supplementary Figure 1.** ^1^H NMR (400 MHz, CD_3_OD) spectrum of verbascoside.

**
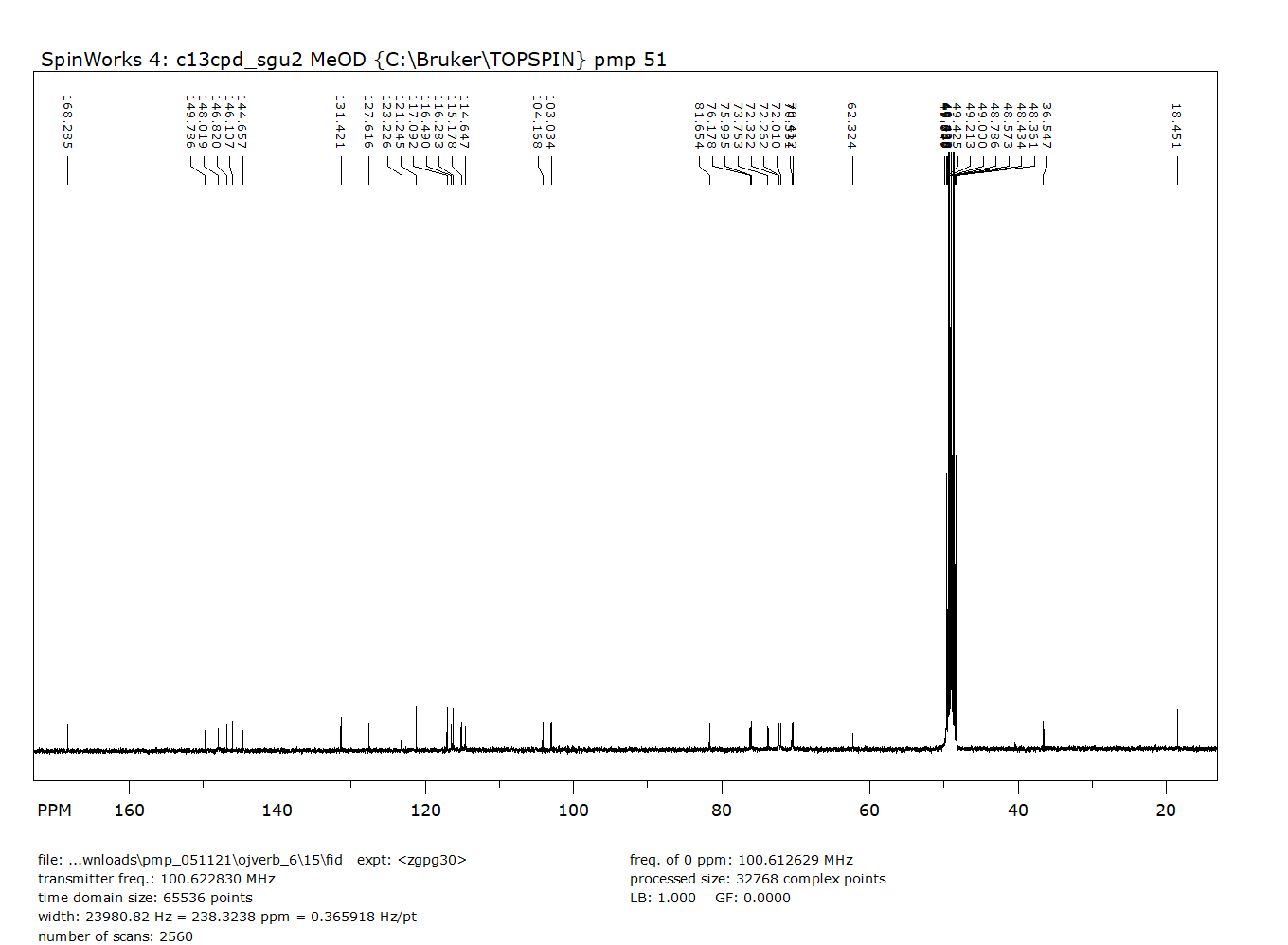
**

**Supplementary Figure 2.** ^13^C NMR (100 MHz, CD_3_OD) spectrum of verbascoside.

**
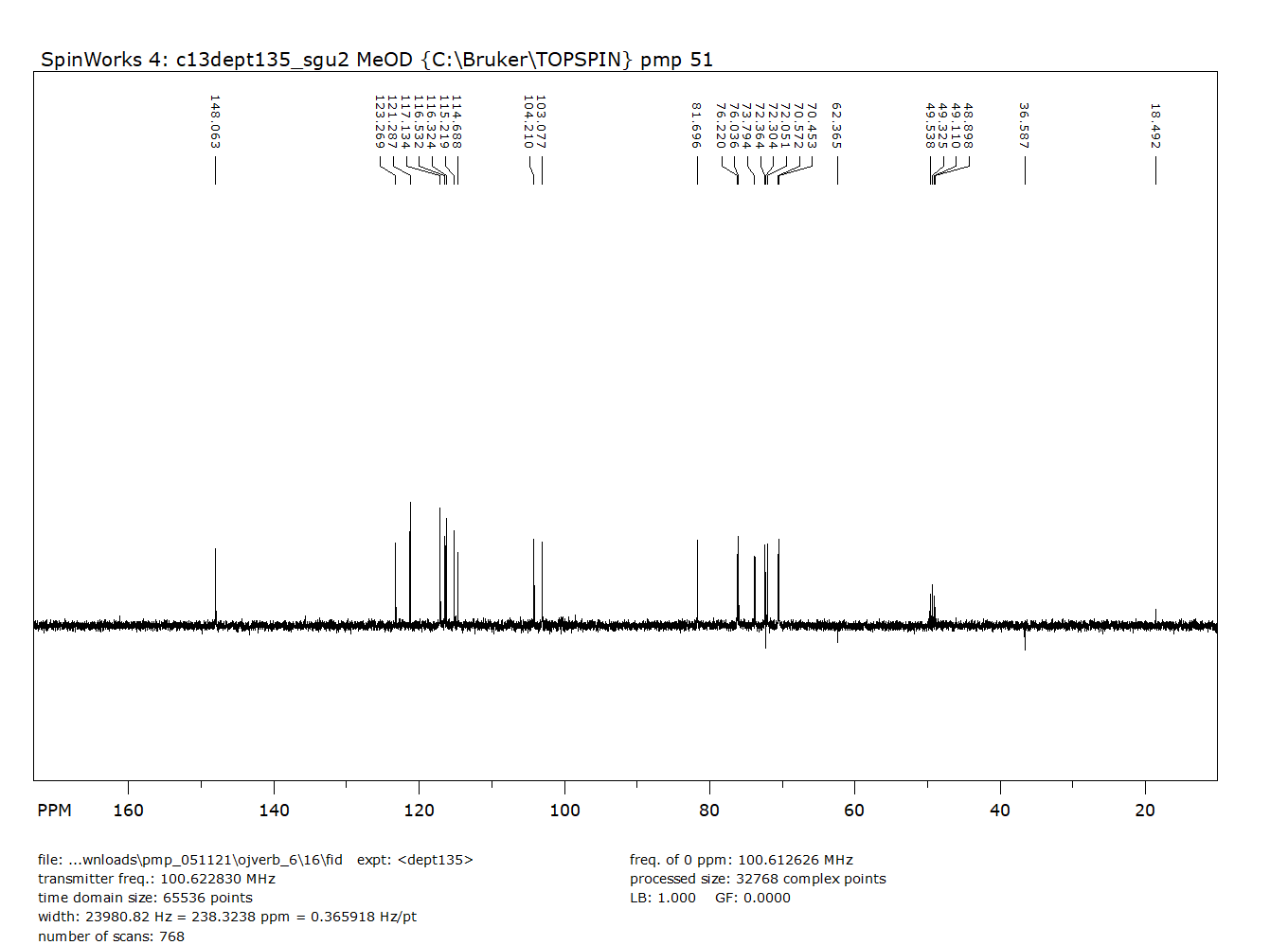
**

**Supplementary Figure 3.** DEPT (100 MHz, CD_3_OD) spectrum of verbascoside.


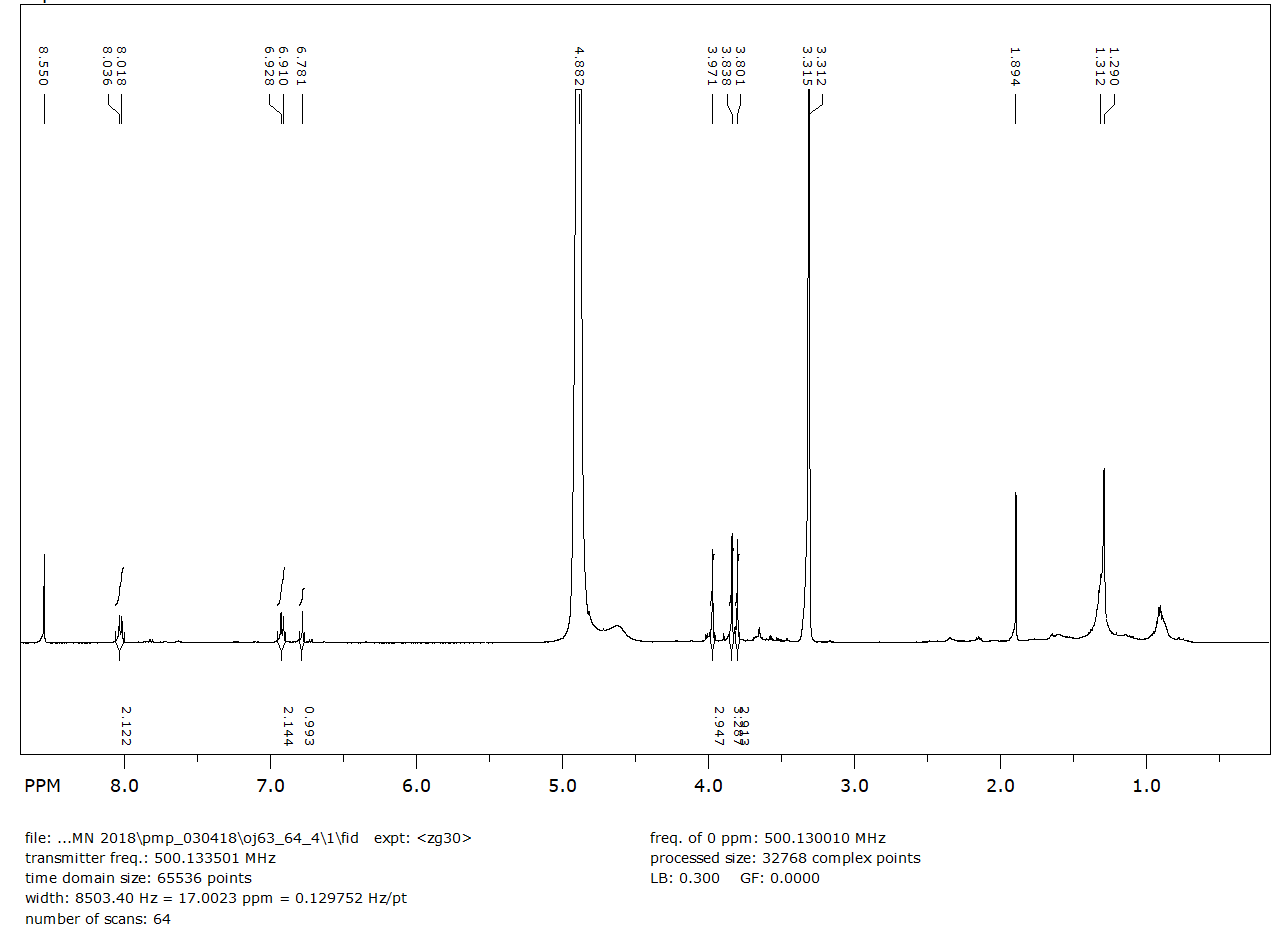


**Supplementary Figure 4.** ^1^H NMR (500 MHz, CD_3_OD) spectrum of penduletin (**2**).


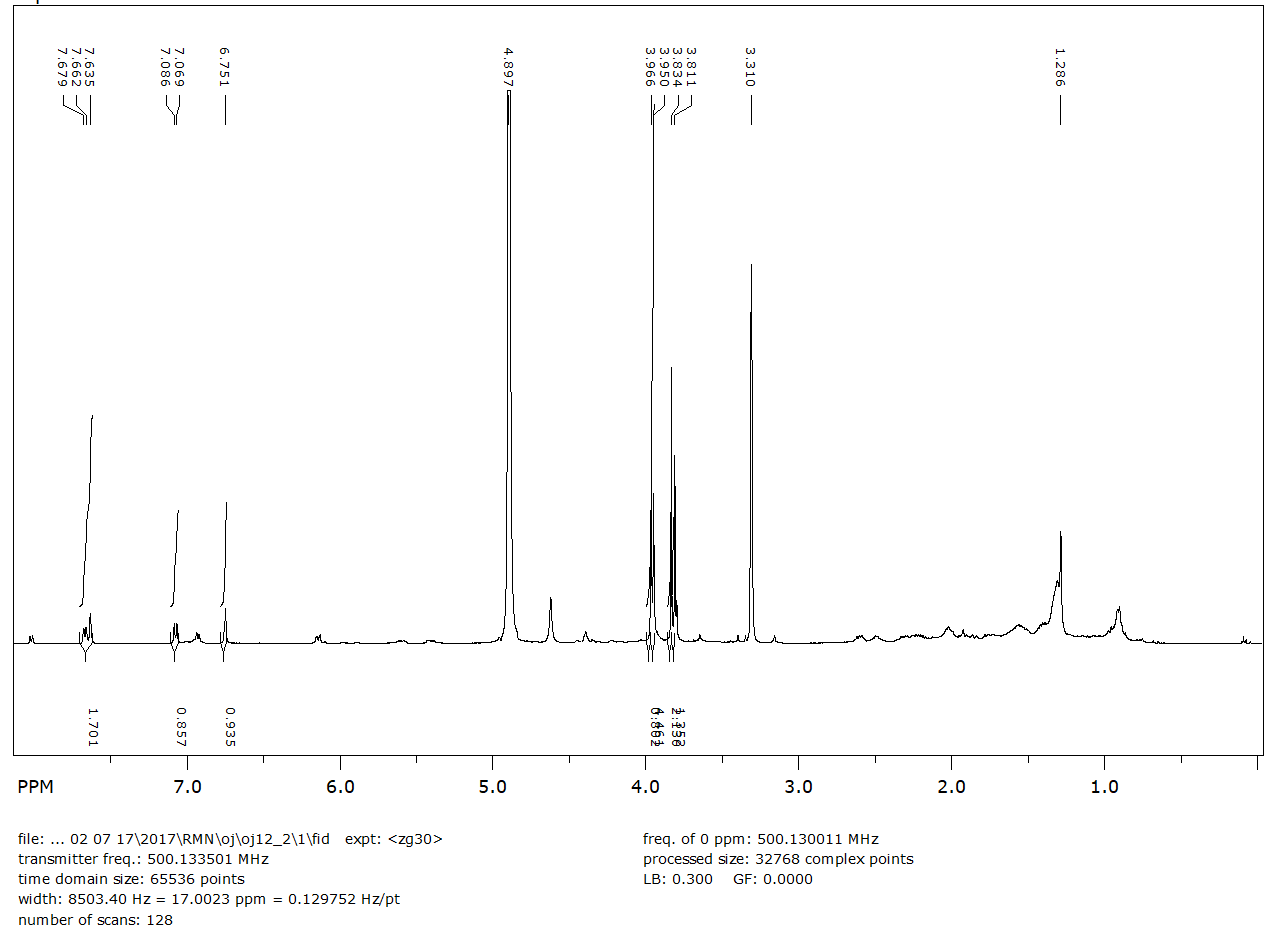
**Supplementary Figure 5.** ^1^H NMR (500 MHz, CD_3_OD) spectrum of casticin (**3**).

**
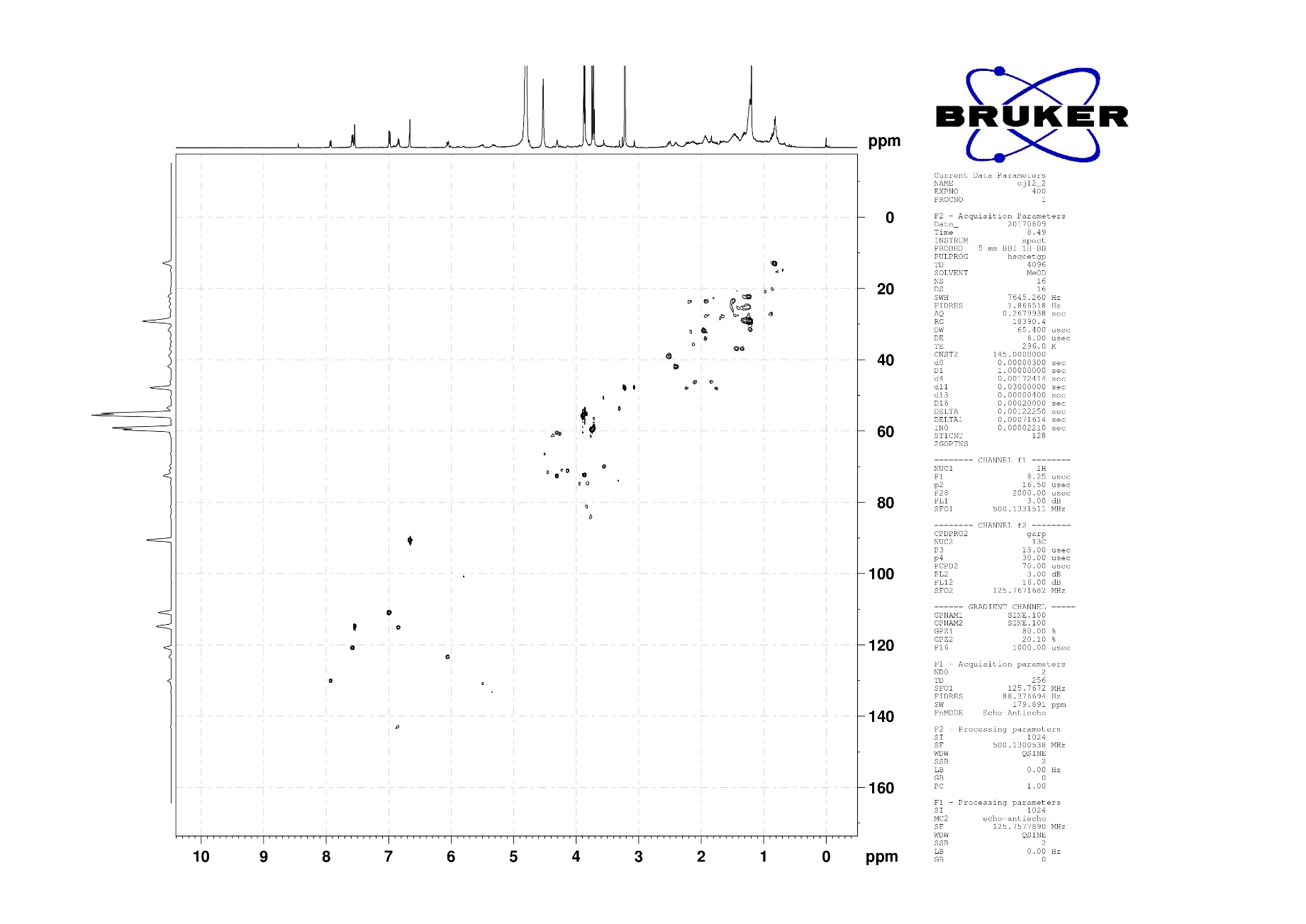
Supplementary Figure 6.** HSQC (500 and 125 MHz, CD_3_OD) spectrum of casticin (**3**).

**
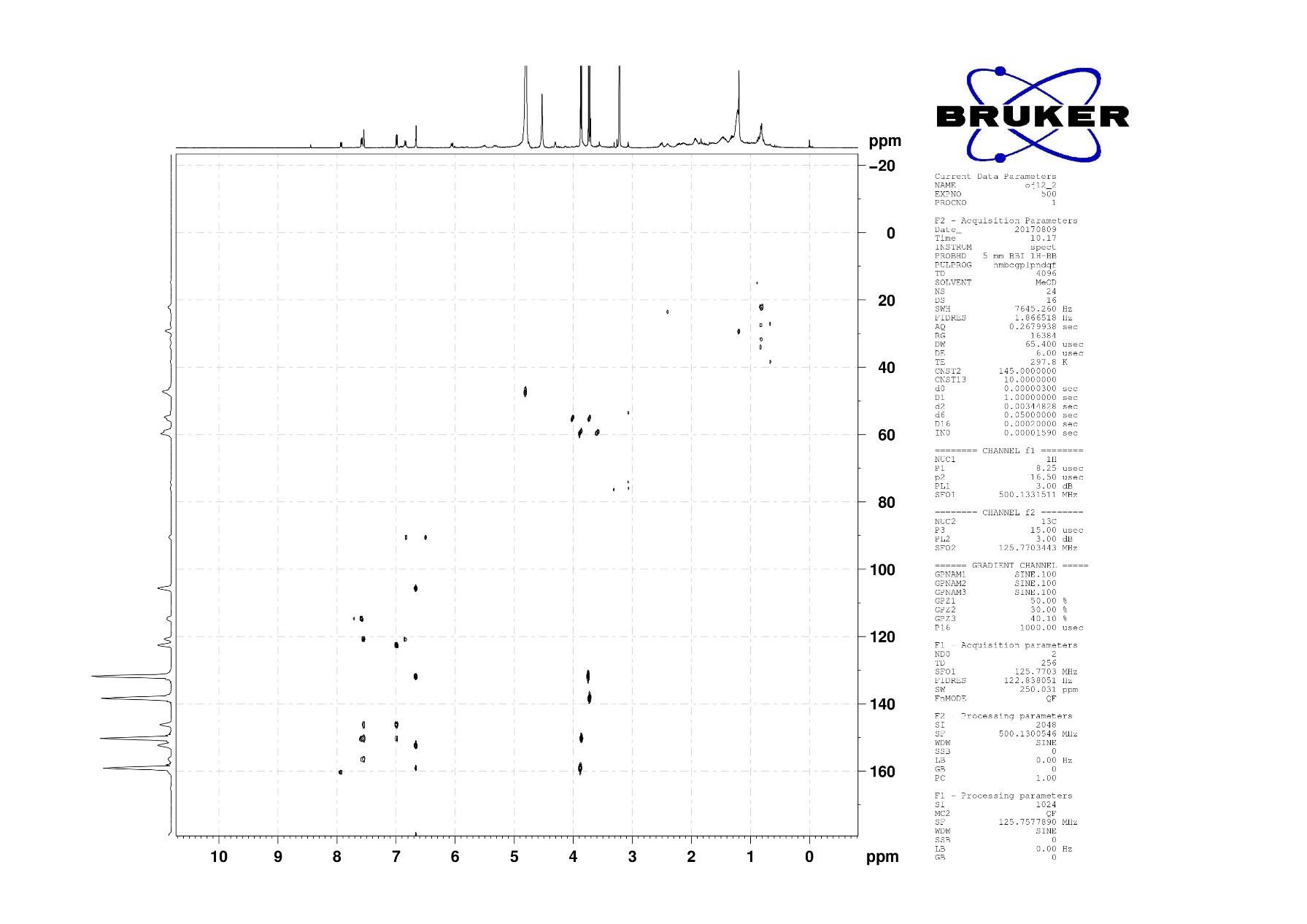
Supplementary Figure 7.** HMBC (500 and 125 MHz, CD_3_OD) spectrum of casticin (**3**).


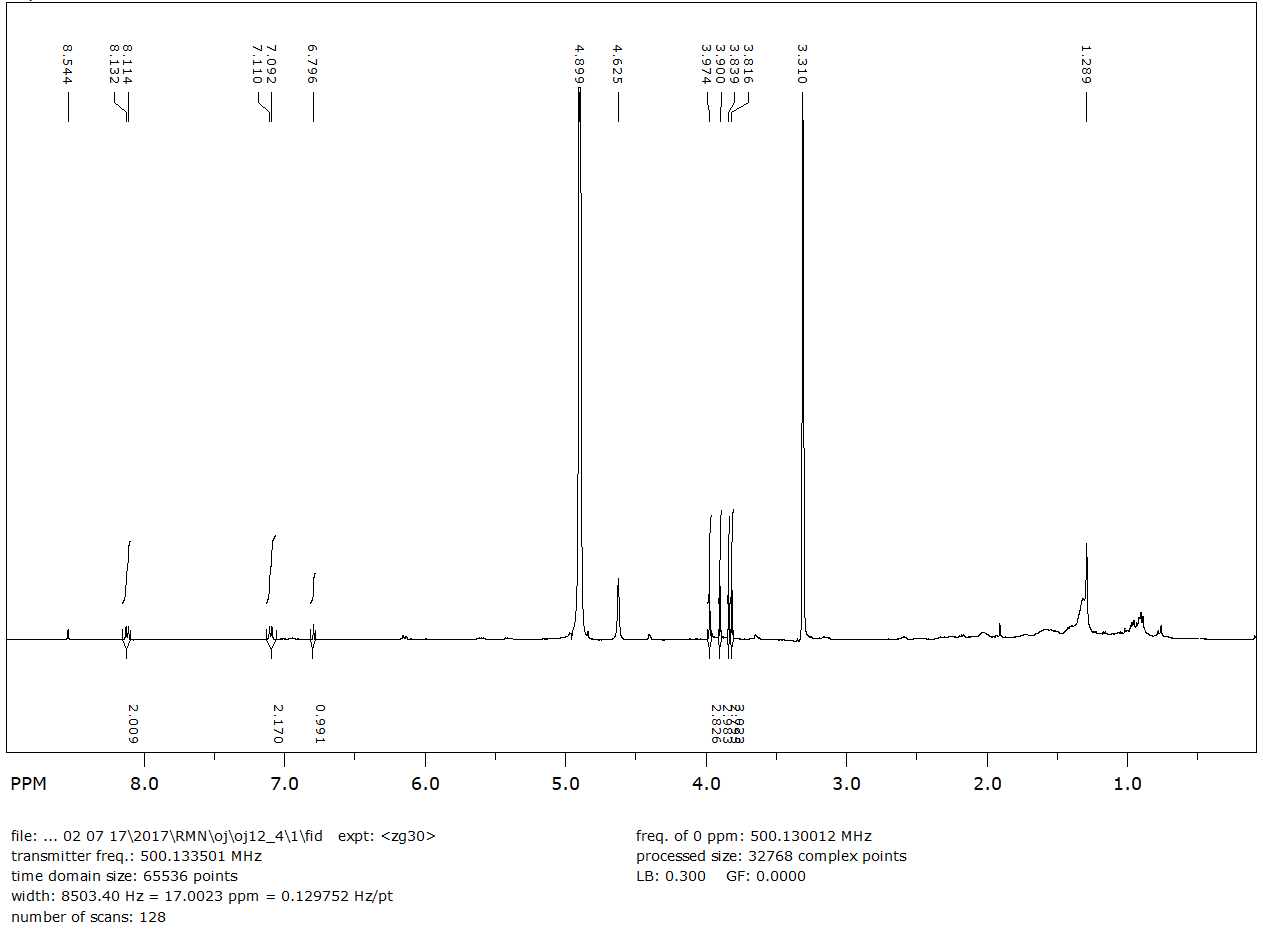


**Supplementary Figure 8.** ^1^H NMR (500 MHz, CD_3_OD) spectrum of penduletin 4ʹ-methyl ether (**4**).

**
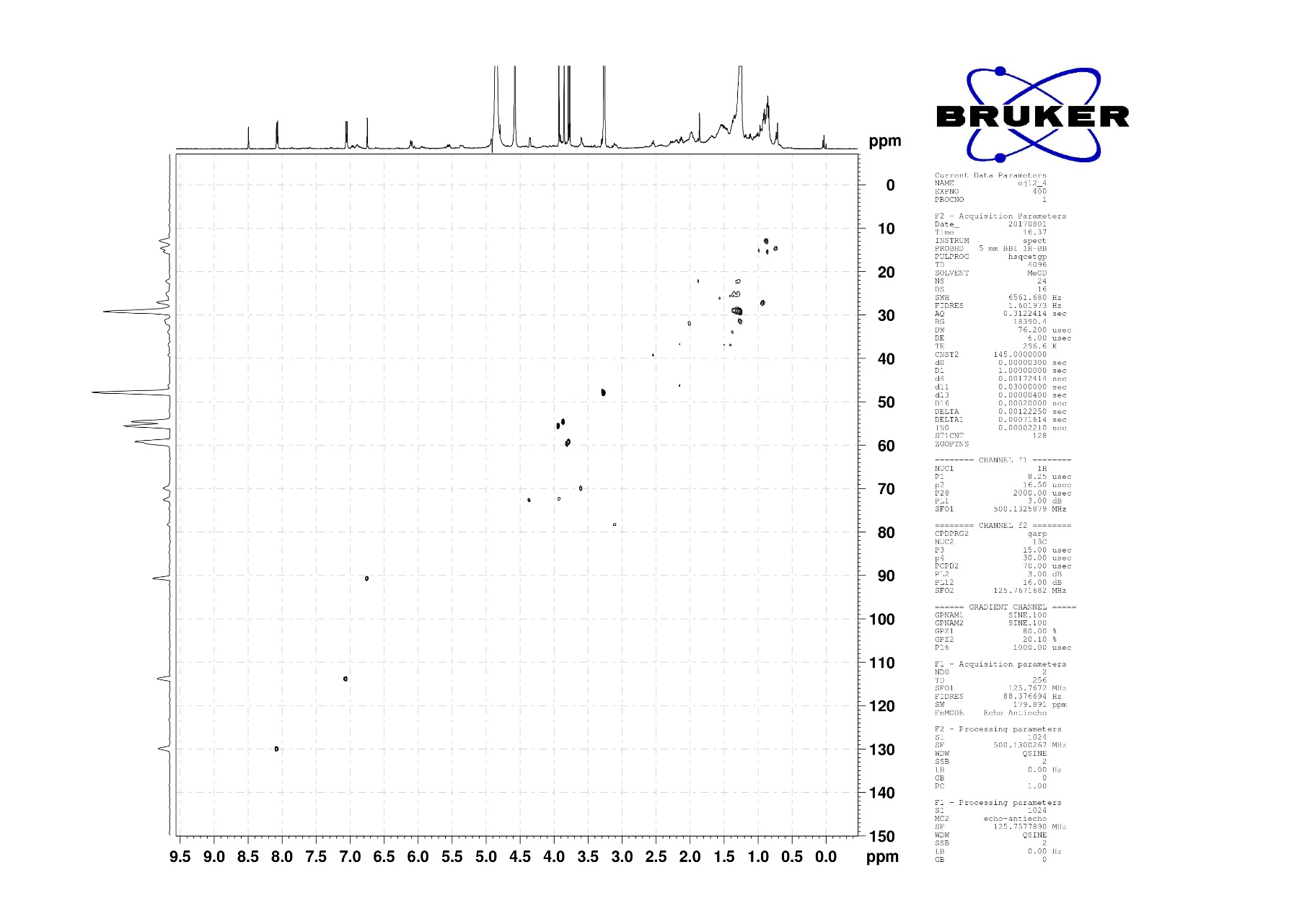
Supplementary Figure 9.** HSQC (500 and 125 MHz, CD_3_OD) spectrum of penduletin 4ʹ-methyl ether (**4**).

**
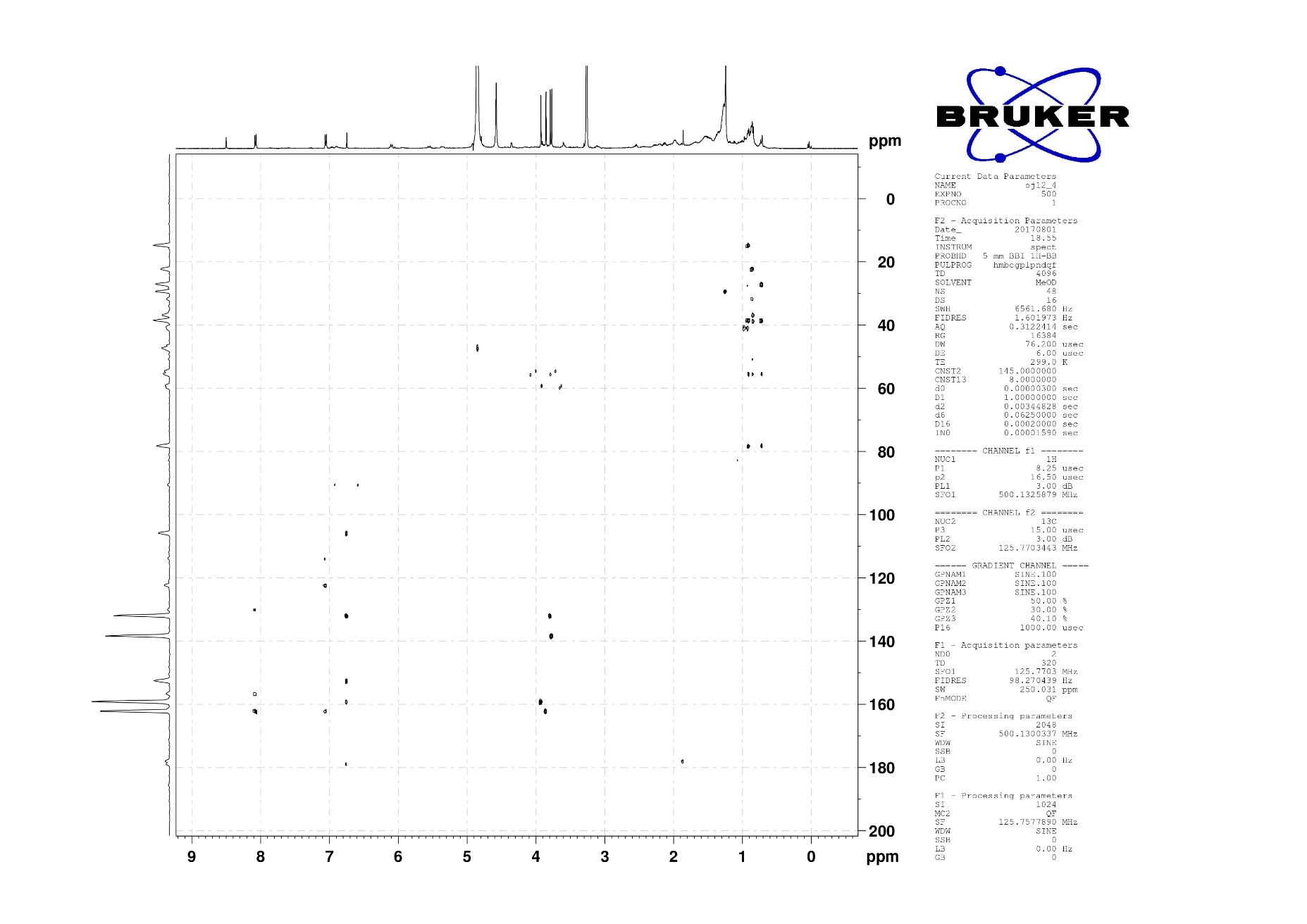
Supplementary Figure 10.** HMBC (500 and 125 MHz, CD_3_OD) spectrum of penduletin 4ʹ-methyl ether (**4**).

**
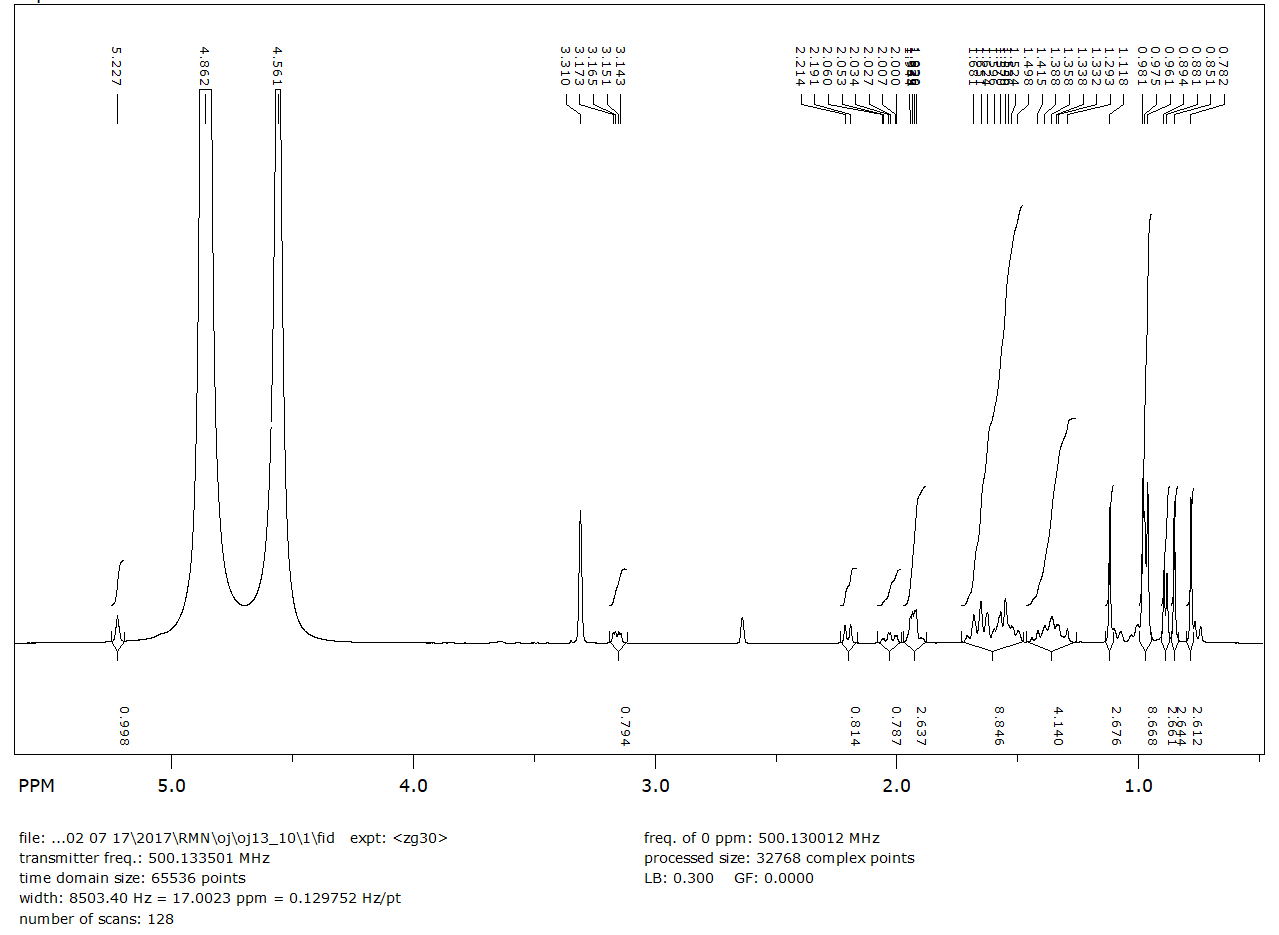
Supplementary Figure 11.** ^1^H NMR (500 MHz, CD_3_OD) spectrum of ursolic acid (**5**).

**
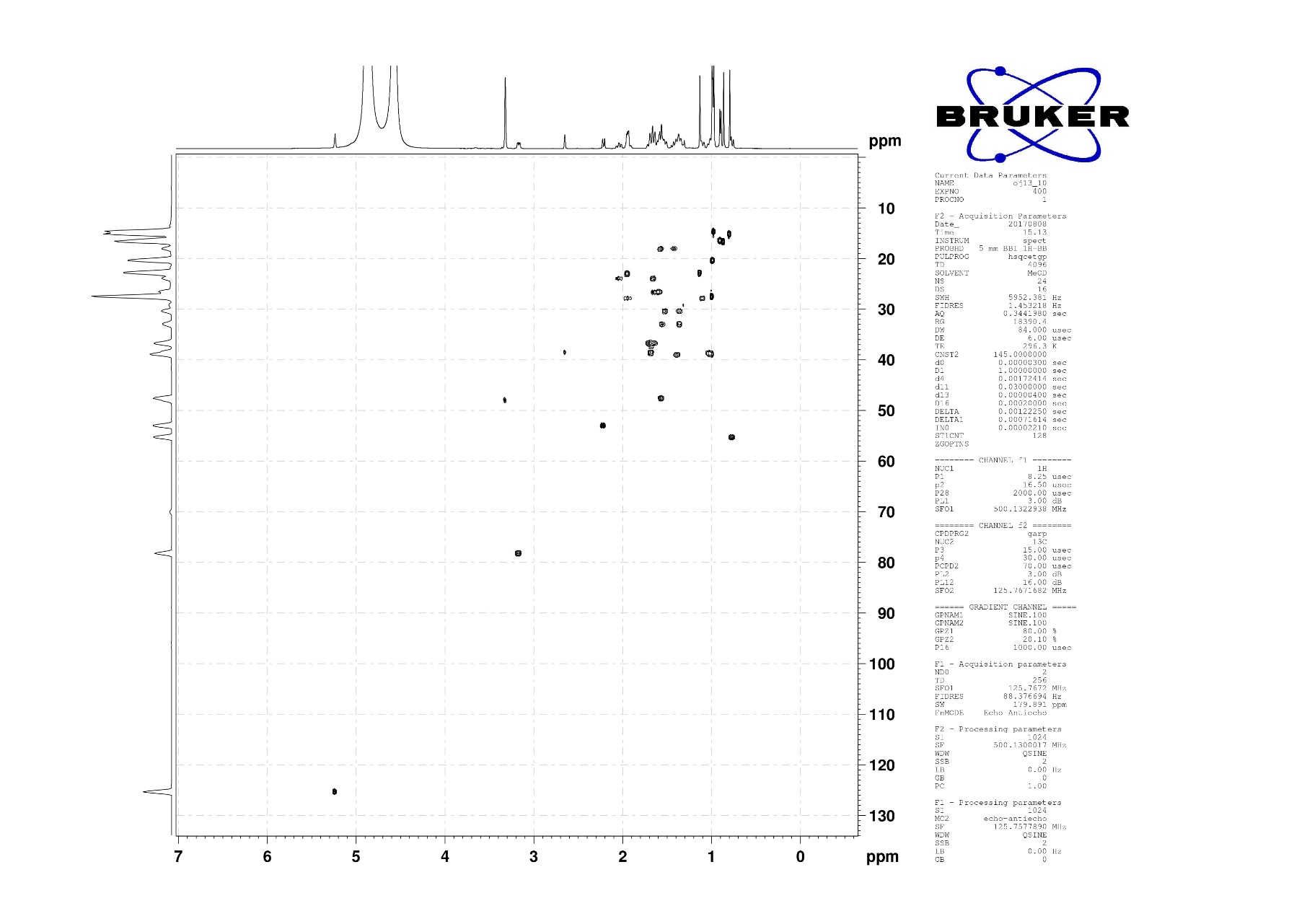
Supplementary Figure 12.** HSQC (500 and 125 MHz, CD_3_OD) spectrum of ursolic acid (**5**).

**
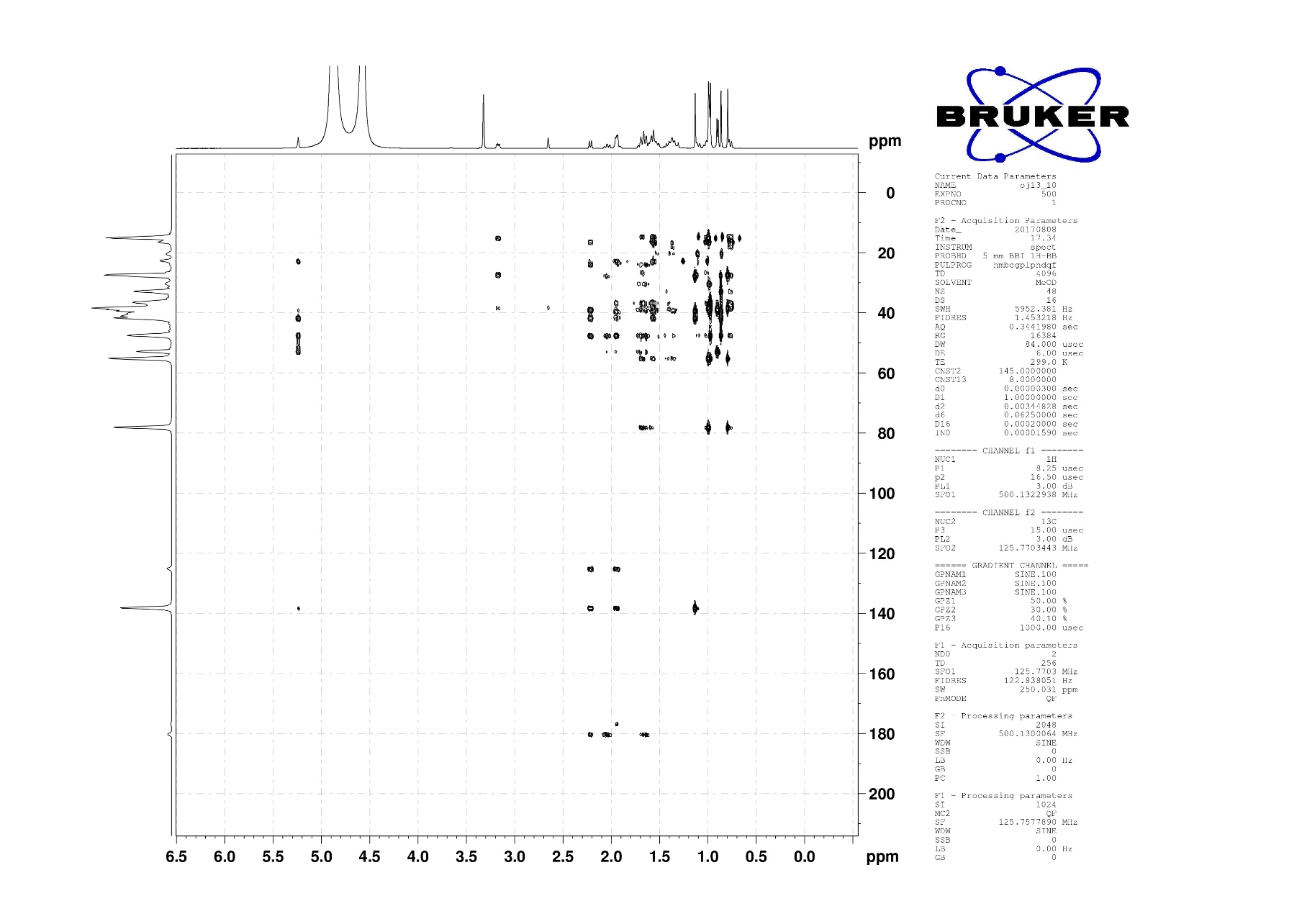
Supplementary Figure 13.** HMBC (500 and 125 MHz, CD_3_OD) spectrum of ursolic acid (**5**).

**
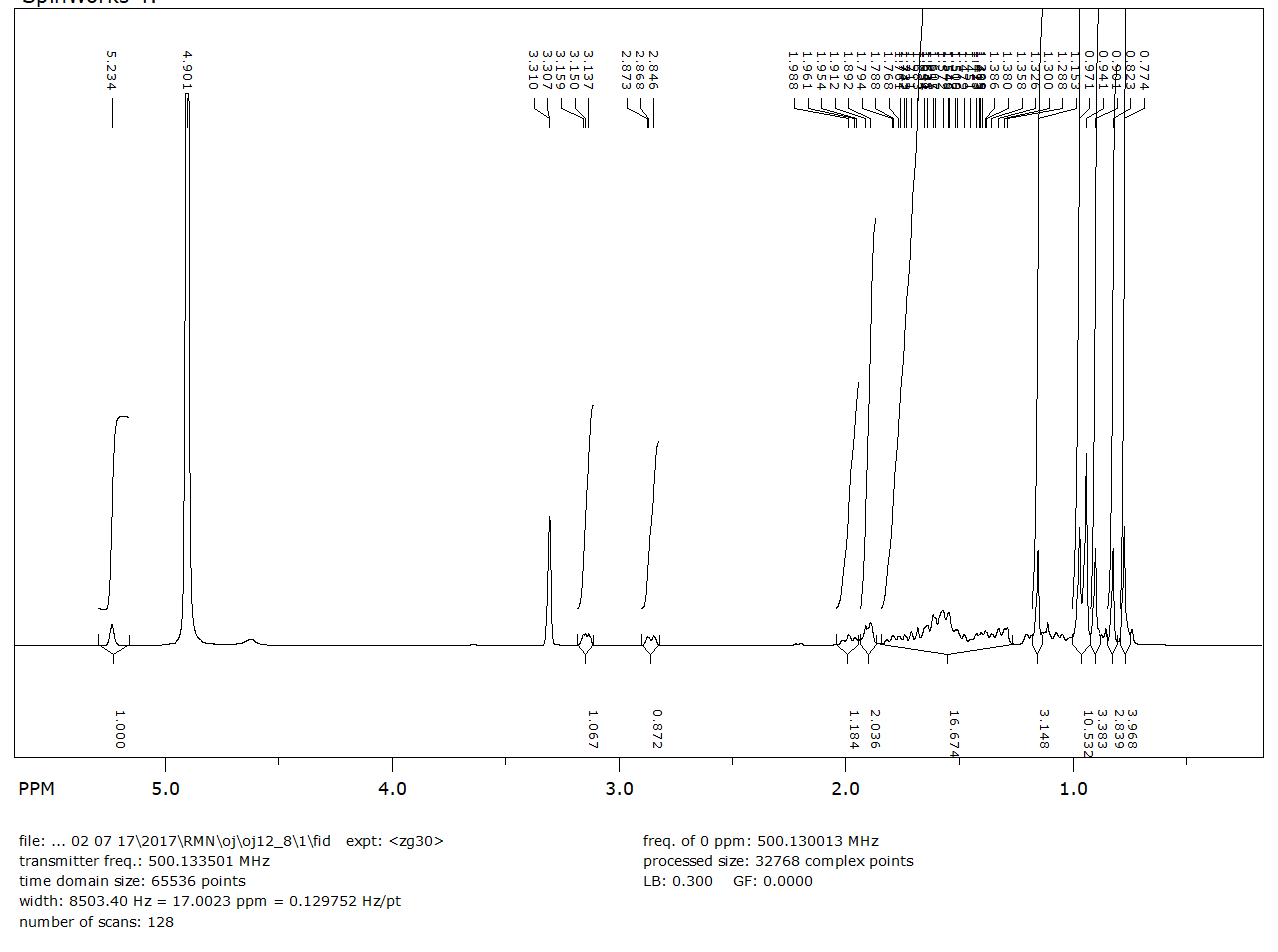
Supplementary Figure 14.** ^1^H NMR (500 MHz, CD_3_OD) spectrum of oleanolic acid (**6**).

**
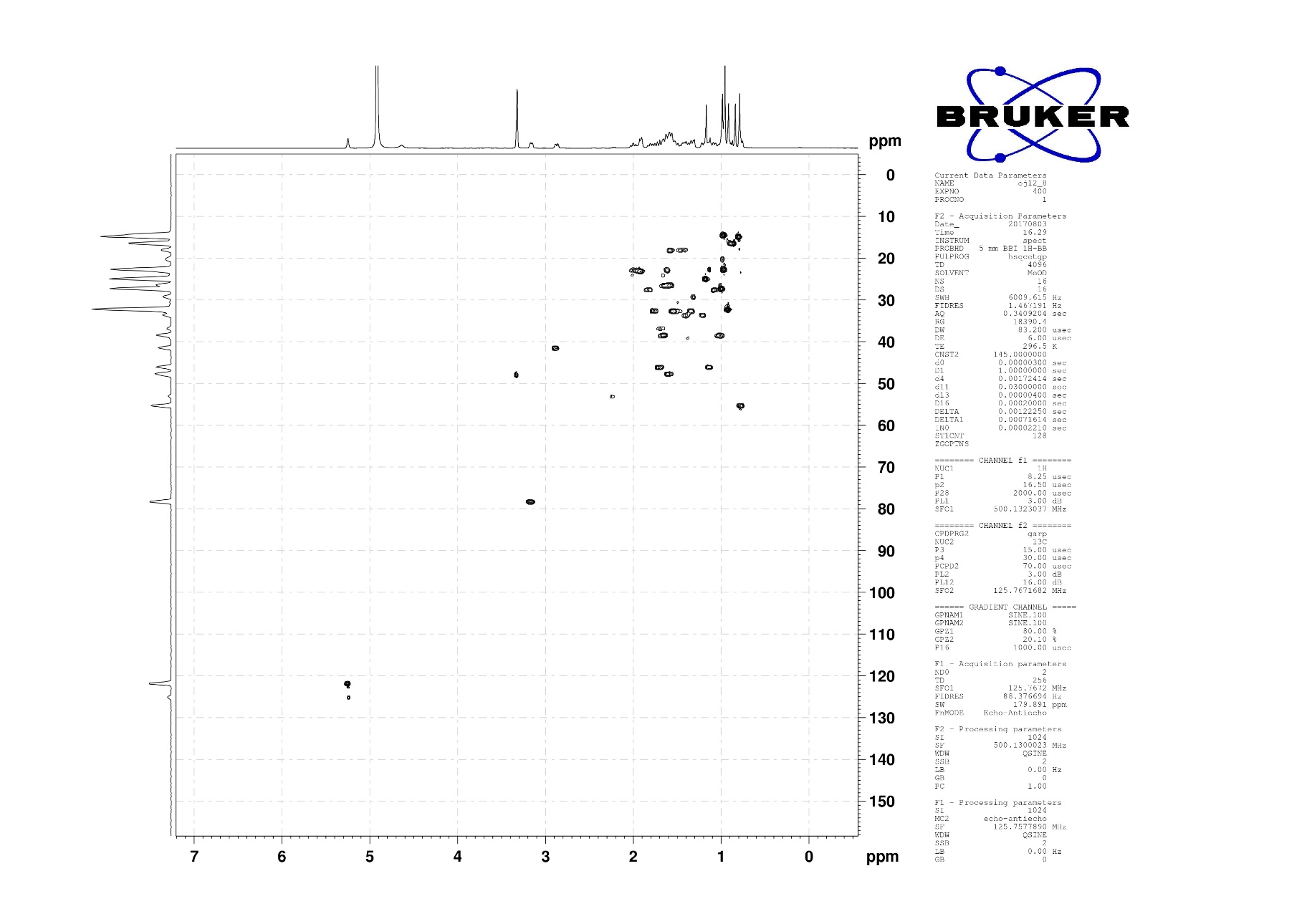
Supplementary Figure 15.** HSQC (500 and 125 MHz, CD_3_OD) spectrum of oleanolic acid (**6**).

**
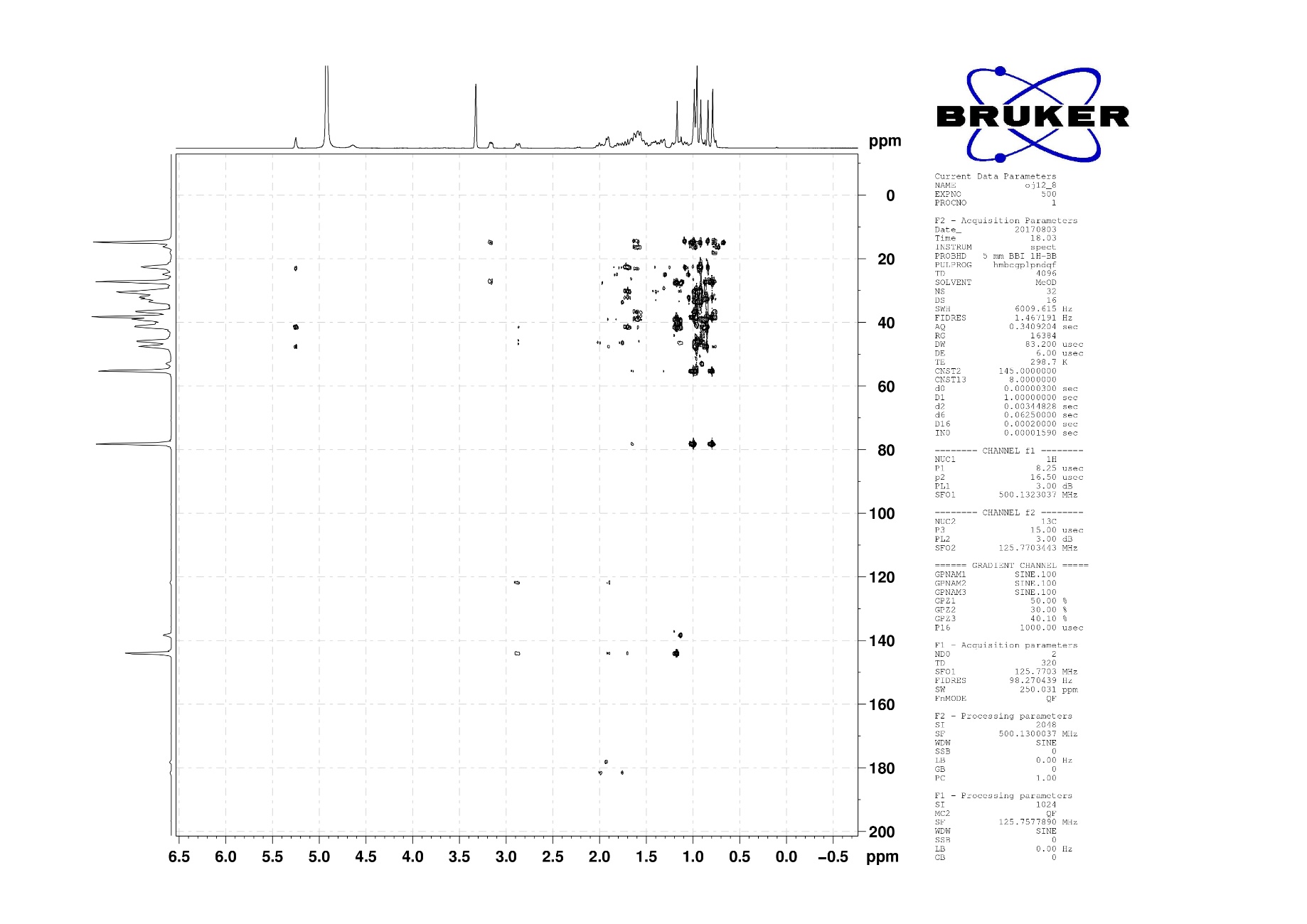
Supplementary Figure 16.** HMBC (500 and 125 MHz, CD_3_OD) spectrum of oleanolic acid (**6**).

**
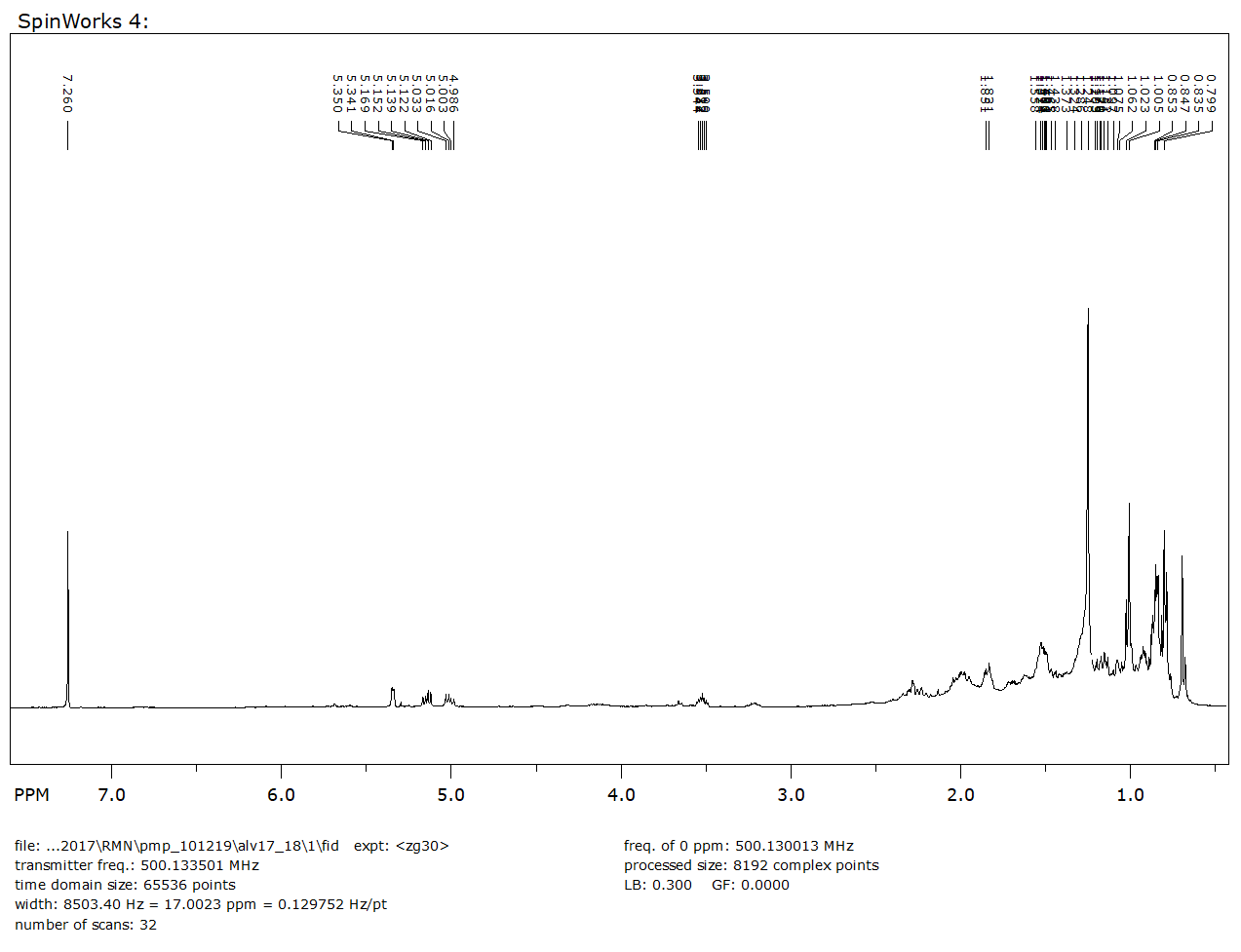
Supplementary Figure 17.** ^1^H NMR (500 MHz, CDCl_3_) spectrum of sitosterol (**7**) and stigmasterol (**8**).

**
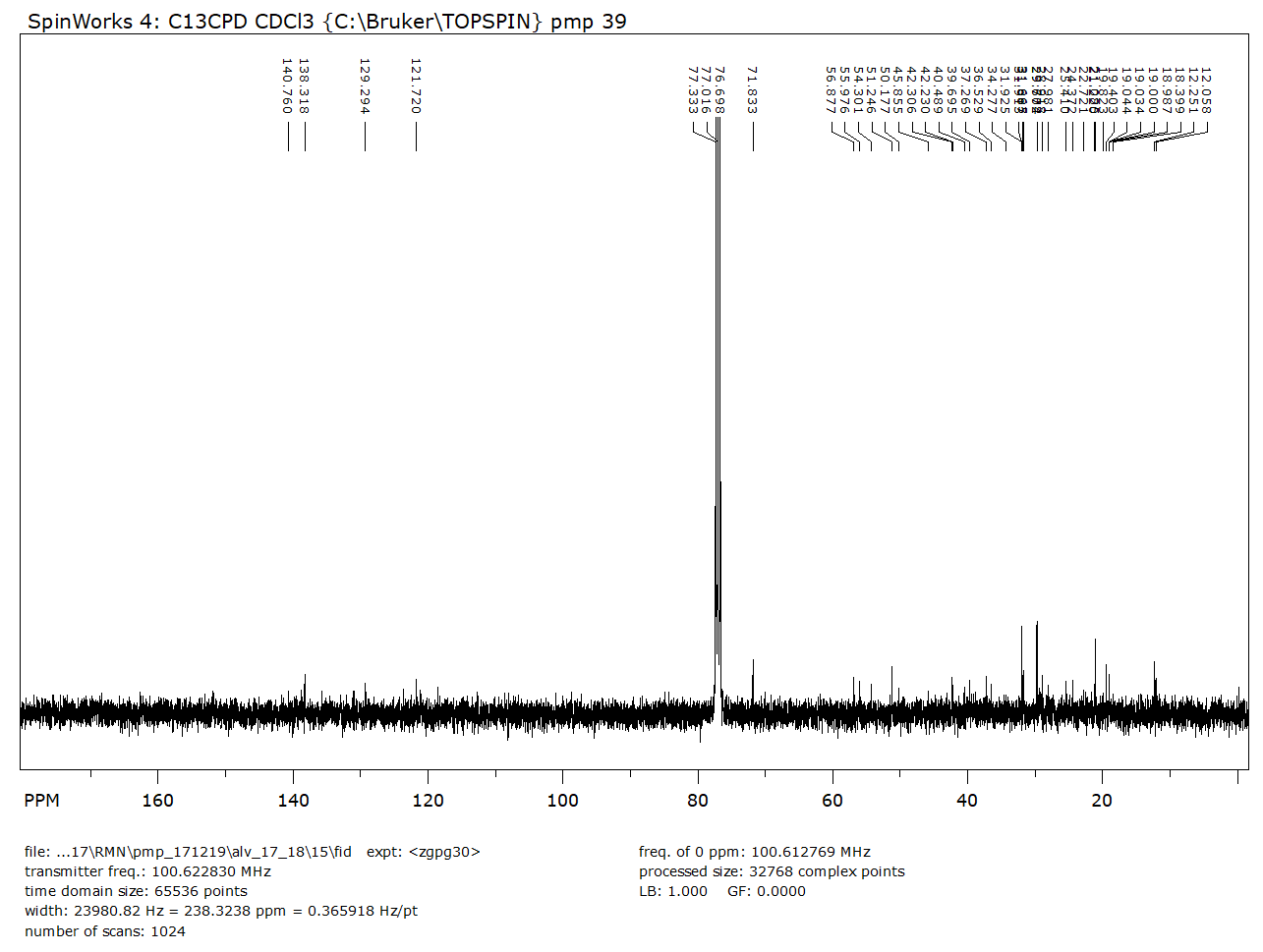
**

**Supplementary Figure 18.** ^13^C NMR (100 MHz, CDCl_3_) spectrum of sitosterol (**7**) and stigmasterol (**8**).

(a)

(b)

(c)

(d)

(e)

(f)

(g)

(h)

**Supplementary Figure 19.** UV spectra and Mass spectra negative mode from the crude extract sample CE-1 (a) t_R_ 10.5 min, (b) t_R_ 10.9 min, (c) t_R_ 11.7 min, (d) t_R_ 12.0 min, (e) t_R_ 12.3 min (f) t_R_ 22.5 min, (g) t_R_ 22.9 min, and (h) t_R_ 23.4 min.

(a)

(b)

(c)

(d)

(e)

(f)

**Supplementary Figure 20.** UV spectra and Mass spectra positive mode from the crude extract sample CE-1 (a) t_R_ 10.5 min, (b) t_R_ 11.7 min, (c) t_R_ 22.5 min, (d) t_R_ 22.9 min, (e) t_R_ 23.4 min, and (f) t_R_ 27.9 min.

(a)

(b)

(c)

(d)

(e)

(f)

(g)

(h)

**Supplementary Figure 21.** UV spectra and Mass spectra negative mode from the crude extract sample CE-2 (a) t_R_ 9.2 min, (b) t_R_ 10.5 min, (c) t_R_ 10.8 min, (d) t_R_ 11.7 min, (e) t_R_ 14.2 min, (f) t_R_ 16.6 min, (g) t_R_ 17.2 min, and (h) t_R_ 23.7 min.

(a)

(b)

(c)

(d)

(e)

(f)

(g)

(h)

(i)

**Supplementary Figure 22.** UV spectra and Mass spectra positive mode from the crude extract sample CE-2 (a) t_R_ 9.2 min, (b) t_R_ 10.5 min, (c) t_R_ 10.8 min, (d) t_R_ 11.7 min, (e) t_R_ 14.2 min, (f) t_R_ 14.9 min, (g) t_R_ 16.6 min, (h) t_R_ 17.2 min, and (i) 23.7 min.
